# Restrained Mitf-associated autophagy by Mulberroside A ameliorates osteoclastogenesis and counteracts OVX-Induced osteoporosis in mice

**DOI:** 10.1038/s41420-024-01847-1

**Published:** 2024-02-15

**Authors:** Hong Xue, Zhenhua Feng, Putao Yuan, Li Qiao, Qiliang Lou, Xiangde Zhao, Qingliang Ma, Shiyu Wang, Yang Shen, Huali Ye, Jiao Cheng, Jiying Wang, Shuanglin Wan, Boya Zhang, Peihua Shi, Xuewu Sun

**Affiliations:** 1 Department of Orthopaedic Surgery, Sir Run Run Shaw Hospital, Zhejiang University School of Medicine, Hangzhou, China; 2 Key Laboratory of Musculoskeletal System Degeneration and Regeneration Translational Research of Zhejiang Province, Hangzhou, China

**Keywords:** Macroautophagy, Osteoporosis

## Abstract

Bone and mineral metabolism homeostasis accounts for the maintenance of normal skeletal remodeling. However, with aging and changes in hormone levels, over-activated osteoclasts disrupt homeostasis, induce osteoporosis, and even cause osteoporotic fractures, leading to an enormous economic burden. Despite the rapid development of pharmacological therapy for osteoporosis, safer and more effective treatments remain to be explored. Here, we demonstrate that Mulberroside A (Mul-A), a natural component extracted from mulberry bark and branches, effectively suppresses osteoclastogenesis in vitro and counteracts bone loss caused by ovariectomy (OVX). The mechanism underlying this effect involves the repression of autophagic flux during osteoclastogenesis by Mul-A, which can be attributed to the restrained expression of microphthalmia-related transcription factor (Mitf) and its nuclear translocation. Importantly, Mitf overexpression partially reverses the inhibitory effects of Mul-A on autophagy and osteoclastogenesis. Moreover, applying two autophagy agonizts, rapamycin and Torin 1, attenuates the osteoclastogenic regulatory role of Mul-A. Collectively, our study demonstrates that Mul-A damages osteoclast differentiation and ameliorates osteoporosis caused by estrogen deficiency by modulation of Mitf-associated autophagy, indicating its therapeutic potential against osteoporosis.

## Introduction

Bone is known for its rigidity and self-healing ability. In physiological situations, rational skeletal remodeling maintains structural integrity [1]. However, under certain pathological conditions such as aging, estrogen deficiency, and drug abuse, this process is disrupted, leading to osteoporosis, a metabolic bone disorder distinguished by declined bone mineral density, bone mass, bone toughness, and increased bone fragility [2]. These microstructural changes significantly increase the risk of fracture. Notably, >75% of osteoporotic fractures occur in postmenopausal women, causing disability and increasing global health expenditures [3]. Despite the rapid development of pharmacological therapies for osteoporosis, their application is limited to varying degrees because of side effects [4–7]. Accordingly, further elucidation of the pathogenesis of osteoporosis and the exploration of novel pharmacotherapies are urgently required.

Bone and mineral homeostasis, a delicate balance maintained by bone resorption and formation, accounts for physiological bone metabolism [8, 9]. Mature multinucleated osteoclasts, the only cell type with bone resorption capacity, are derived from haematopoietic progenitors, and their overactivation is considered the primary cause of osteoporosis in postmenopausal women. The receptor activator of nuclear factor-κB (NF-κB) ligand (RANKL) and macrophage colony-stimulating factor (M-CSF) govern the maturation and destiny of osteoclasts [10, 11]. M-CSF is involved in the viability and growth of osteoclast precursor cells [12, 13], whereas RANKL initiates osteoclast differentiation by binding to RANK [14–16]. After combining with RANKL, RANK recruits TRAF6 and activates a series of downstream intricate signaling pathways, such as mitogen-activated protein kinases (MAPKs), nuclear factor-κB (NF-κB), calcium signaling pathways, and phosphatidylinositol 3-kinase (PI3K) which further drive the activation of nuclear factor-activated T cells 1 (Nfatc1) to promote osteoclastogenesis [17]. The osteoclast-specific genes, including cathepsin K (Ctsk), tartrate-resistant alkaline phosphatase (Trap), and dendritic cell-specific transmembrane protein (Dc-stamp), are subsequently upregulated to facilitate the osteoclast maturation and function [18].

Autophagy, referred to as type II cell death, is a cellular process regulated by autophagy-related genes (Atgs), in which cells degrade macromolecules and damaged organelles through lysosomes [19]. The ubiquitin protein system, mTOR signaling pathway, and various transcription factors, including Tfeb, Tfe3, and Mitf, regulate autophagy [20–22]. Irregularity of autophagy is correlated with many diseases, such as cancer, neurodegenerative diseases, and microbial infections [23]. In addition, the relationship between autophagy and degenerative skeletal diseases has recently attracted considerable attention. Single nucleotide polymorphisms in autophagy-related genes have been reported to be closely associated with height or bone mineral density [24]. Furthermore, the autophagy-related proteins Atg5, Atg7, and Lc3 have been identified to participate in osteoclastogenesis. Atg5^fl/fl^; Lyz2-Cre mice can counteract the ovariectomy (OVX) induced bone loss compared to Atg5^fl/fl^ mice [25]. Atg7 deficiency suppresses Trap and Ctsk expression in osteoclasts [26]. Moreover, the mutual transformation of Lc3I and Lc3II also involves osteoclast activity [27]. Therefore, autophagy presents as a plausible therapeutic target for treating osteoclast-associated osteoporosis.

Mulberroside A, a stilbene glycoside compound extracted from mulberry bark and rhizomes, exhibits various pharmacological effects, including anti-inflammatory, antioxidant, and nerve protection [28–31]. Mul-A has also been proven to attenuate the activation of NF-kB and MAPKs and reduce Mitf expression [32, 33]. Therefore, it is reasonable to assume that Mul-A may be involved in the regulation of osteoclasts and may have potential healing benefits for osteoporosis. In our study, we conducted various experiments in vitro and constructed an OVX animal model to simulate osteoporosis caused by estrogen deficiency. The purpose was to verify the impact of Mul-A on osteoclastogenesis and explore its underlying mechanisms.

## Results

### Mul-A suppresses the diffrentiation of BMMs in vitro

**Fig. 1:**
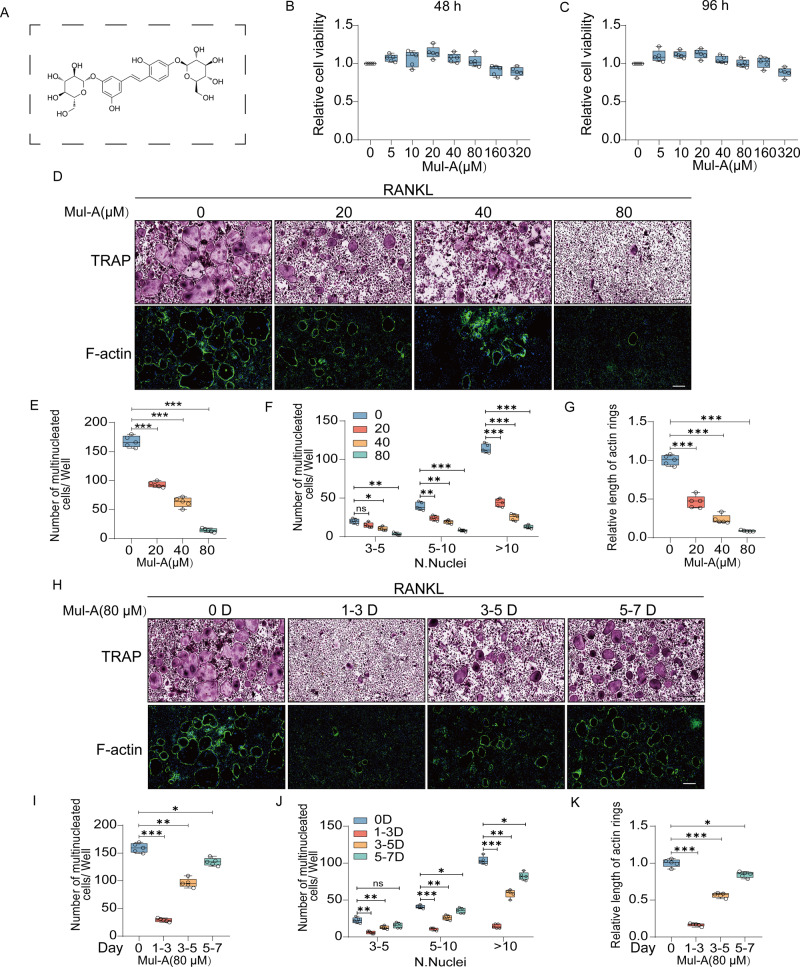
Mul-A suppresses the diffrentiation of BMMs in vitro. **A** The chemical structure of Mul-A. **B**, **C** The activity of BMMs was assessed at different concentrations of Mul-A. **D** BMMs were cultured with 0, 20, 40, and 80 μM Mul-A for 6 days under the conditions of 30 ng/mL M-CSF and 50 ng/mL RANKL. Double staining with Trap and phalloidin was performed (*n* = 5). **E**–**G** Quantitative analysis was conducted to evaluate the number of Trap-positive multinuclear cells and the length of F-actin per well (*n* = 5). **H** BMMs were treated with 80 μM Mul-A in the early (1-3 D), middle (3-5 D), and late (5-7 D) stages of osteoclast differentiation. Double staining with Trap and phalloidin was performed (*n* = 5). **I**–**K** Quantitative analysis was performed to measure the number of Trap-positive multinuclear cells and the length of F-actin per well (*n* = 5). Scale bar = 200 μm. The control group was treated with an equivalent amount of DMSO. Data were presented as the median and interquartile range (IQR). *P < 0.05, **P < 0.01, ***P < 0.001.

The chemical structure of Mul-A is shown in Fig. 1A. A CCK8 assay was performed at 48 and 96 h to examine the toxicity of Mul-A on BMMs. As shown, the activity of BMMs was not significantly changed by Mul-A at concentrations below 320 μM (Fig. 1B, C). Given the slightly altered activities of BMMs at 160 μM, 80 μM was selected as the maximum concentration for subsequent experiments.

During the osteoclast differentiation, Mul-A was confirmed to reduce the number of TRAP^+^ multinuclear cells and the length of F-actin in a concentration-dependent way with the aid of Trap and F-actin staining (Fig. 1D–G). Both decreased shapely in the presence of 80 μM Mul-A. Moreover, Mul-A was applied during the indicated osteoclastogenesis phases to identify the stage at which it functioned. As shown, Mul-A disturbed the entire process of osteoclastogenesis, especially in the early stages (1-3 Days) (Fig. 1H–K). These results demonstrate that Mul-A impairs osteoclast maturation without affecting proliferation.

### Mul-A attenuates the bone resorption capacity and specific gene expression of osteoclasts

**Fig. 2:**
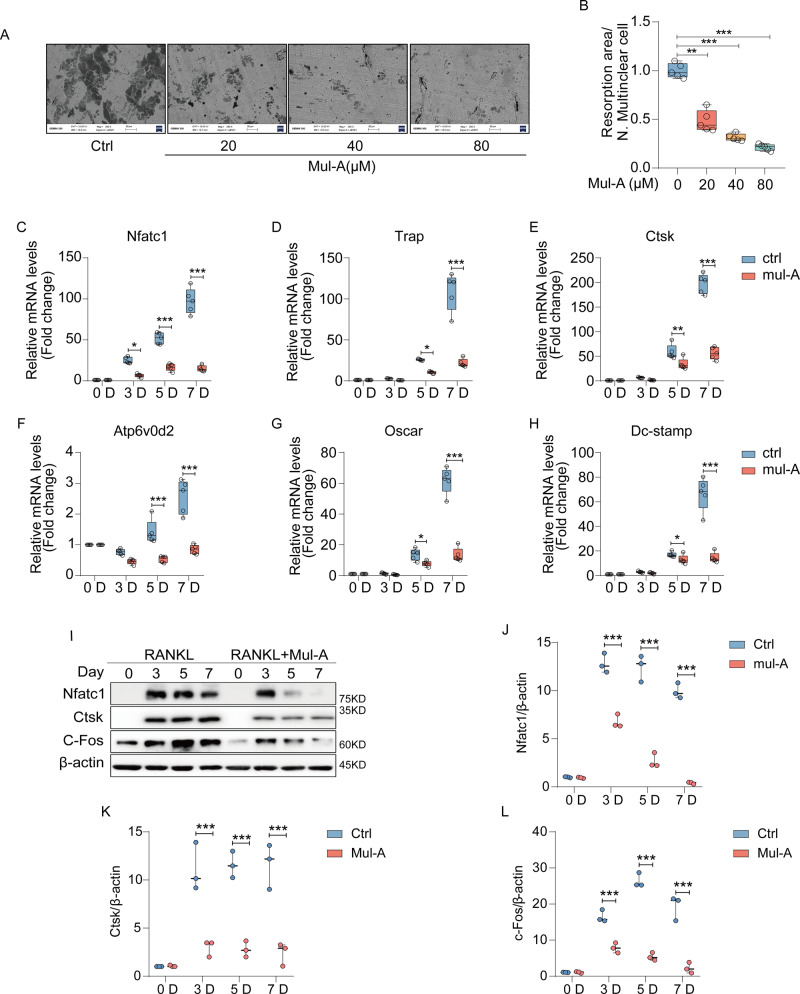
Mul-A attenuates the bone resorption capacity and specific gene expression of osteoclasts. **A** BMMs were cultured with 30 ng/mL M-CSF and 50 ng/mL RANKL for 6 days. Following the maturation of osteoclasts, cells were subsequently treated with various concentrations of Mul-A (0, 20, 40, 80 μM) for an additional 4-5 days. The bovine bone slices were collected for scanning electron microscopy analysis. **B** Quantitative analysis of bone resorption capacity per cell (*n* = 5). **C**–**H** BMMs were treated with 50 ng/mL RANKL and 30 ng/mL M-CSF in the presence or absence of 80 μM Mul-A for 0, 3, 5, and 7 days. The mRNA levels of osteoclast-related genes were quantitatively analyzed using quantitative real-time PCR (*n* = 5). **I** BMMs were treated with 50 ng/mL RANKL and 30 ng/mL M-CSF in the presence or absence of 80 μM Mul-A for 0, 3, 5, and 7 days. The control group was treated with the equivalent amount of DMSO. The protein levels of Nfatc1, Ctsk, and C-Fos were examined by western blotting. **J**–**L** Quantitative evaluation of the relative grayscale intensity of protein bands (*n* = 3). Scale bar = 50 μm. The control group was added with an equivalent DMSO. Data were presented as the median and interquartile range (IQR). *P < 0.05, **P < 0.01, ***P < 0.001.

Osteoclasts secrete HCL to form an acidic microenvironment that exerts bone resorption capacity to maintain bone remodeling [34]. However, under certain pathological conditions, the enhanced bone resorption of osteoclasts tends to induce osteoporosis. Thus, a bone resorption experiment was used to evaluate the impact of Mul-A on osteoclast absorption. The results showed a significant reduction in the size and number of absorption pits as the dosage of Mul-A increased. This indicates that Mul-A has the potential to suppress osteoclastic bone resorption (Fig. 2A, B).

Osteoclast-specific genes such as Nfatc1 play non-fungible roles in osteoclast maturation. Accordingly, quantitative real-time PCR and Western blotting were performed to determine whether Mul-A impaired mRNA and protein expression, respectively. Osteoclast-specific genes induced by RANKL decreased following treatment with Mul-A in a dose- and time-dependent manner (Fig. 2C–H, Supplementary Fig. 1A–F). Similar results were obtained using Western blotting (Fig. 2I–L, Supplementary Fig. 1G–K). Thus, in addition to inhibiting maturation, Mul-A exerts regulatory effects on bone resorption and osteoclast-specific gene expression.

### Mul-A suppresses the autophagy flux occurrence during osteoclastogenesis

**Fig. 3:**
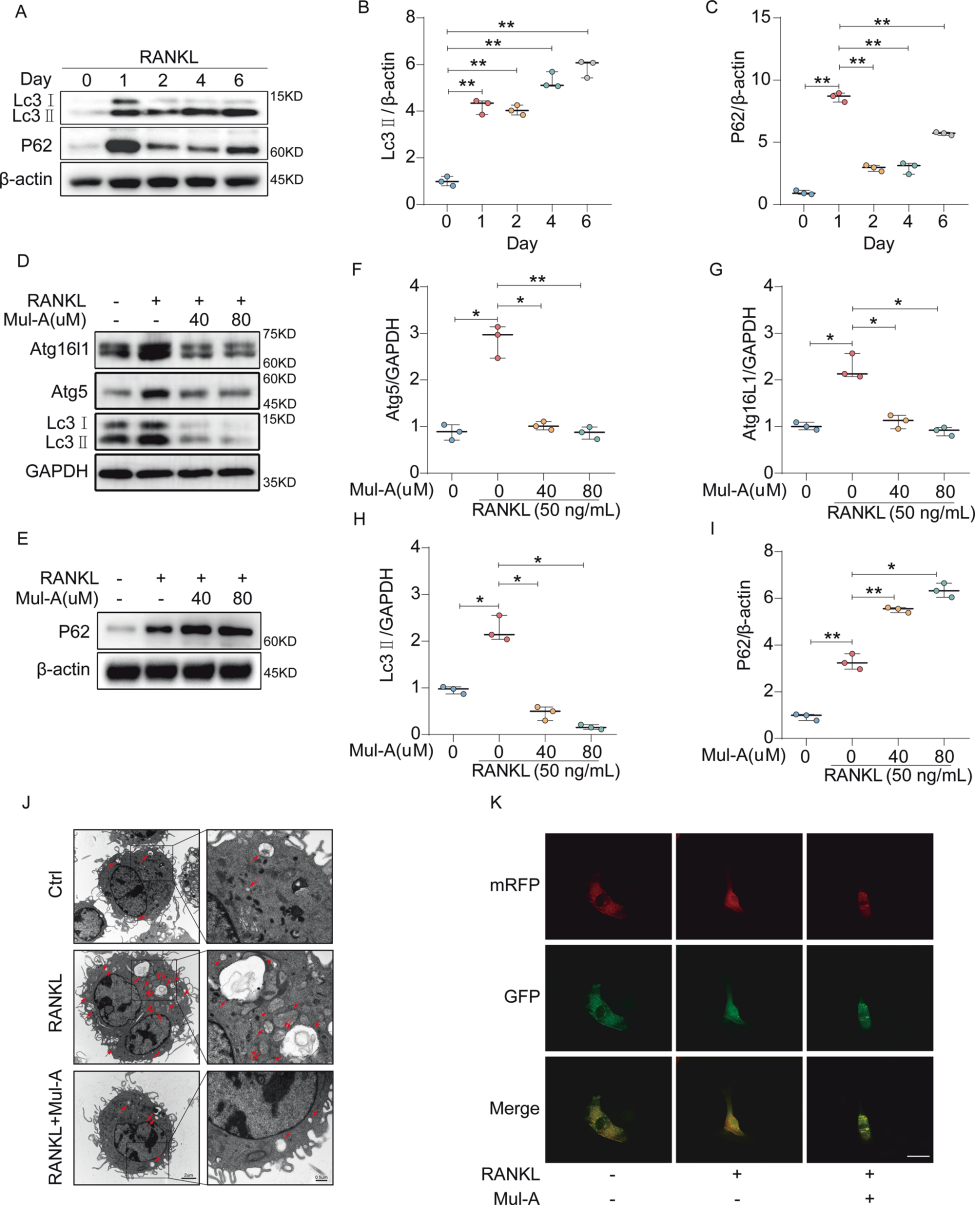
Mul-A suppresses the occurrence of autophagy flux during osteoclastogenesis. **A** BMMs were stimulated with 50 ng/mL RANKL and 30 ng/mL M-CSF for 0, 1, 2, 4, and 6 days. Cell lysates were extracted for western blot analysis to assess the expression of Lc3 and P62 proteins (*n* = 3). **B**, **C** Quantitative analysis of the relative grayscale intensity of Lc3 and P62 bands (*n* = 3). **D**, **E** BMMs were treated with 50 ng/mL of RANKL, 30 ng/mL of M-CSF, and different concentrations of Mul-A (0, 40, 80 μM) for 4 days. Cell lysates were then extracted for western blot analysis of autophagy-related proteins (*n* = 3). **F**–**I** Quantitative analysis of the relative grayscale intensity of autophagy-related protein bands (*n* = 3). **J** BMMs were treated with 50 ng/mL of RANKL and 30 ng/mL M-CSF for 4 days in the presence or absence of Mul-A, and the cell microstructure was fixed using 2.5% glutaraldehyde. Autophagosomes and lysosomes were observed using transmission electron microscopy (*n* = 5). Scale bars = 2 μm, Scale bars = 0.5 μm. **K** BMMs transfected with mRFP-GFP-Lc3 adenovirus were treated with 50 ng/mL of RANKL and 30 ng/mL of M-CSF for 4 days in the presence or absence of Mul-A (80 μM). The cells were fixed in 4% paraformaldehyde for 15 min, and fluorescent spots were observed using confocal microscopy (*n* = 5). Scale bar = 50 μm. The control group was added with an equivalent DMSO. Data were presented as the median and interquartile range (IQR). *P < 0.05, **P < 0.01, ***P < 0.001.

Considering that moderate autophagy is closely related to osteoclasts’ differentiation and absorption properties [35, 36], the effects of Mul-A on the autophagic flux during osteoclastogenesis were further explored. In our study, autophagic flux was minimally observed in the absence of RANKL. The expression of Lc3II increased, with prolonged RANKL treatment, accompanied by decreased P62 expression, indicating that autophagy was activated during osteoclastogenesis (Fig. 3A–C). However, this phenomenon was interrupted by the Mul-A intervention. As presented in Fig. 3D–I, Mul-A eliminated the increase of autophagy-related proteins (Lc3II, Atg16l1, Atg5) and upregulated the expression of P62 induced by RANKL, which was consistent with Quantitative real-time PCR (Supplementary Fig. 2A–F). Morphological changes in autophagosomes and lysosomes were detected using transmission electron microscopy. Our results demonstrated that RANKL stimulation accelerated the fusion of autophagosomes and lysosomes. Mul-A interrupted the fusion process and suppressed autophagosome formation (Fig. 3J). Taking another approach, the mRFP-GFP-Lc3 adenovirus was used to track autophagy. Mul-A treatment repressed RANKL-induced GFP fluorescence quenching, which further confirmed that the fusion of autophagosomes and lysosomes was inhibited by Mul-A (Fig. 3K). Collectively, Mul-A inhibits the activation of autophagy during osteoclastogenesis.

### Mitf partially accounts for the regulation of Mul-A on autophagy

Mitf, a dimeric transcription factor involved in autophagy and lysosomal biogenesis, has been reported to be suppressed by Mul-A in melanocytes [37, 38]. Its expression is closely associated with osteoclastogenesis [39, 40]. Furthermore, Mitf is known to be regulated by the p38 and ERK. Our previous data showed that Mul-A significantly inhibited the phosphorylation levels of p38 and ERK, but not JNK, during RANKL stimulation (Supplementary Fig. 3A–D). Thus, we speculated that Mitf acts as the target of autophagy regulation by Mul-A.

**Fig. 4:**
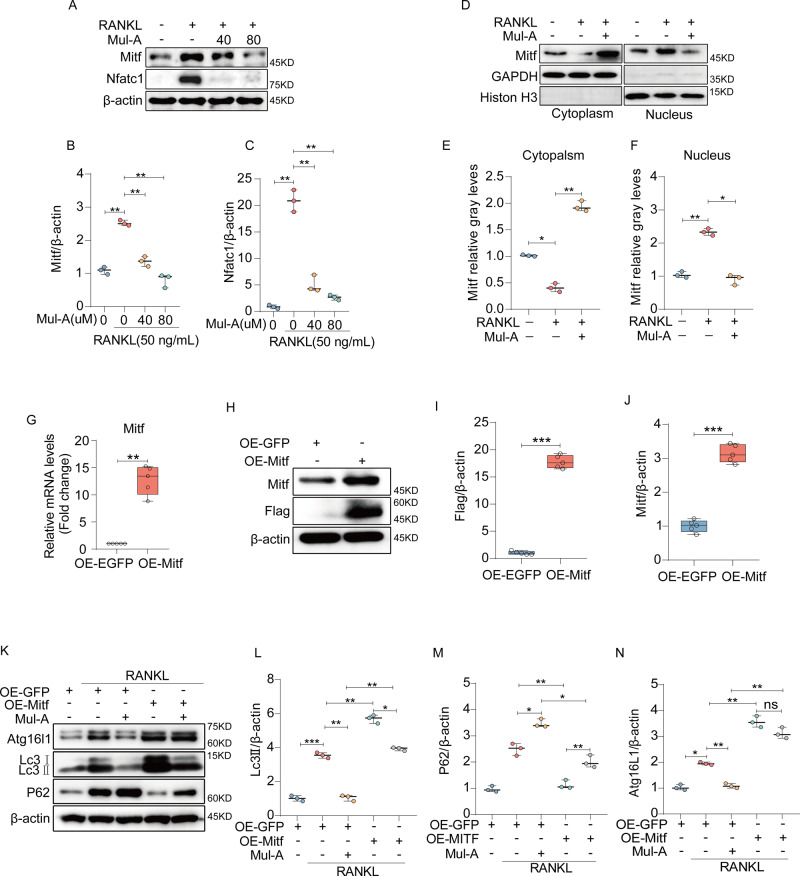
Mitf partially accounts for the regulation of Mul-A on autophagy. **A** BMMs were treated with 50 ng/mL of RANKL and 30 ng/mL of M-CSF in the presence or absence of Mul-A (0, 40, 80 μM) for 4 days. The protein expression of Mitf and Nfatc1 was analyzed using western blotting (*n* = 3). **B**, **C** Quantitative analysis was performed to determine the relative grayscale intensity of the protein bands (*n* = 3). **D** RAW264.7 cells were pretreated with Mul-A (80 μM) for 2 h and then stimulated with 50 ng/mL of RANKL for 30 min. Cytoplasmic and nuclear proteins were isolated to analyze the distribution of Mitf (*n* = 3). **E**, **F** Quantitative analysis was conducted to determine the relative grayscale intensity of Mitf bands (*n* = 3). **G** BMMs were transfected with 3xflag Mitf adenovirus for 2 days, and then mRNA levels of Mitf were analyzed using quantitative real-time PCR (*n* = 5). **H** BMMs were collected to extract whole proteins for western blot analysis of Mitf and Flag after transfection with 3xflag Mitf adenovirus for 2 days (*n* = 3). **I**, **J** Quantitative analysis was performed to determine the relative grayscale intensity of Mitf and Flag (*n* = 3). **K** After transfection with 3xflag Mitf adenovirus, BMMs were continuously treated with 30 ng/mL of M-CSF and 50 ng/mL of RANKL in the presence or absence of Mul-A for 4 days. The expression of autophagy-related proteins was analyzed using western blotting (*n* = 3). **L**–**N** Quantitative analysis was conducted to determine the relative grayscale intensity of the protein bands (*n* = 3). The control group was added with an equivalent DMSO. Data were presented as the median and interquartile range (IQR). *P < 0.05, **P < 0.01, ***P < 0.001.

Firstly, we investigated the impact of Mul-A on Mitf. As confirmed by western blotting, Mitf expression was upregulated upon stimulation with RANKL, while Mul-A incubation reduced its expression in a dose-dependent manner (Fig. 4A–C). In addition, the nuclear translocation of Mitf, which is important for the transcriptional regulation of autophagy-related genes, was restrained by Mul-A (Fig. 4D–F). A 3 × Flag labeled Mitf-overexpressing adenovirus was constructed to validate whether Mitf is the downstream target of Mul-A. Western blotting and Quantitative real-time PCR were conducted to determine the Mitf expression levels after transfection. The Mitf adenovirus significantly upregulated the expression of Mitf compared to that in the GFP (Fig. 4G–J). A subsequent quantitative real-time PCR assay indicated that Mitf overexpression visibly augmented the transcription of autophagy-related genes and reversed the inhibition of Mul-A on these genes (Supplementary Fig. 4A–F). Furthermore, Mitf overexpression accelerated the transformation of LC3I to LC3II and counteracted the inhibitory effect of Mul-A on this process. Correspondingly, the regulation of Atg16L1 and p62 by Mul-A was reversed by Mitf overexpression (Fig. 4K–N). Generally, Mitf partially accounts for the autophagy regulation by Mul-A.

### Mitf overexpression reverses the inhibitory role of Mul-A on osteoclastogenesis

**Fig. 5:**
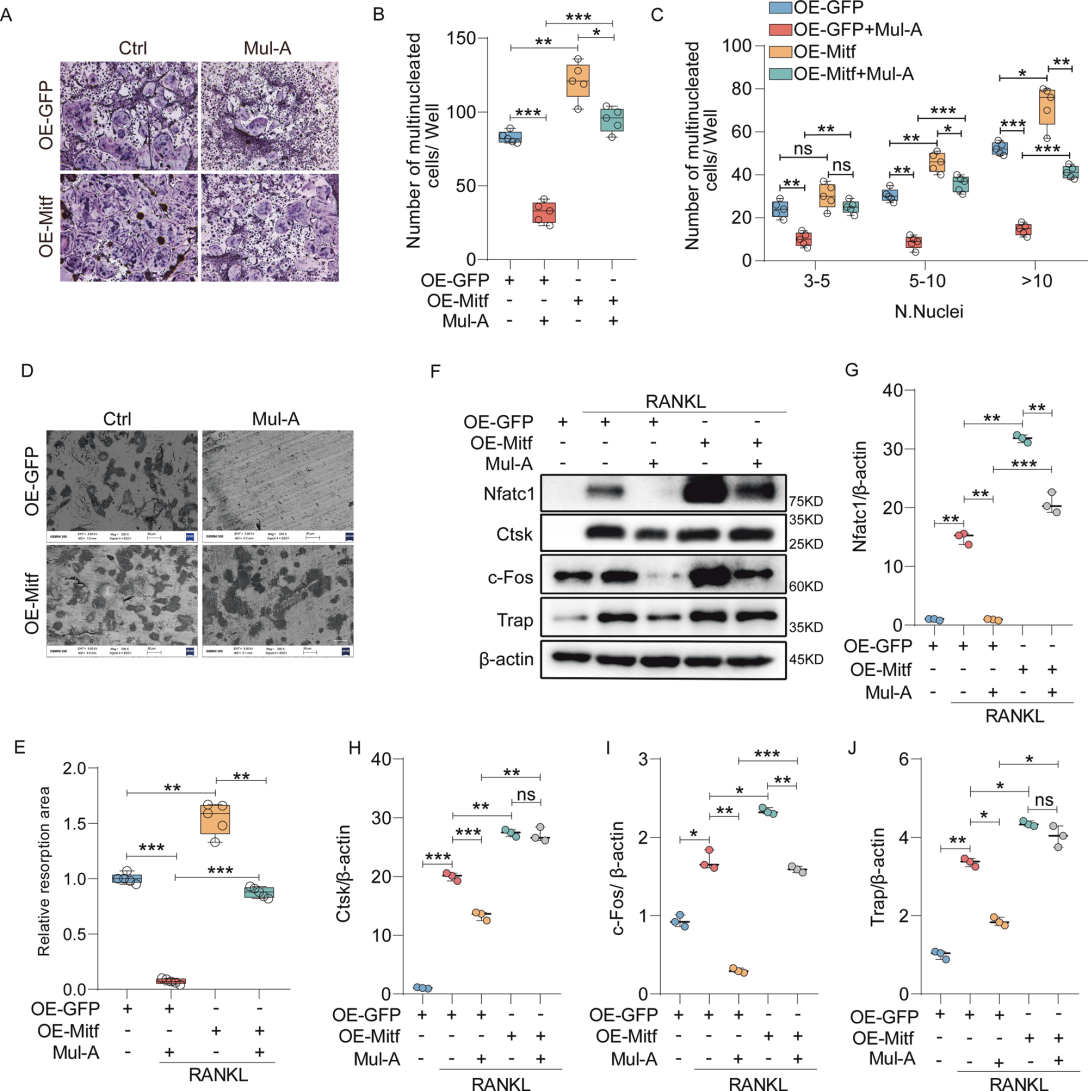
Mitf overexpression reverses the inhibitory role of Mul-A on osteoclastogenesis. **A** BMMs were transfected with Mitf adenovirus for 2 days, followed by continuous treatment with 50 ng/mL of RANKL and 30 ng/mL of M-CSF in the presence or absence of Mul-A for 6 days. Paraformaldehyde fixation was performed, followed by trap staining to visualize the Trap-positive multinuclear cells (*n* = 5). Scale bar = 200 μm. **B**, **C** Quantitative analysis was conducted to determine the number of Trap-positive multinuclear cells per well (*n* = 5). **D** BMMs transfected with Mitf adenovirus for 2 days were subsequently stimulated with 50 ng/mL of RANKL and 30 ng/mL of M-CSF for 6 days. After the formation of mature osteoclasts, cells were treated with or without Mul-A continuously for an additional 4-5 days. Bovine bone slices were collected and analyzed using scanning electron microscopy (*n* = 5). Scale bar = 50 μm. **E** Quantitative analysis was performed to determine the area of bone resorption pits (*n* = 5). **F** BMMs, transfected with Mitf adenovirus, were continuously treated with 50 ng/mL of RANKL and 30 ng/mL of M-CSF in the presence or absence of Mul-A for 5 days. Western blotting was employed to evaluate the expression of osteoclast marker proteins (*n* = 3). **G**–**J** Quantitative analysis was conducted to determine the relative grayscale intensity of the protein bands (*n* = 3). The control group was added with an equivalent DMSO. Data were presented as the median and interquartile range (IQR). *P < 0.05, **P < 0.01, ***P < 0.001.

Since Mitf is involved in the regulation of autophagy by Mul-A, its potential role in osteoclast was further investigated. BMMs were transfected with GFP- or Mitf-overexpressing adenovirus and subjected to different treatments, as indicated. Subsequent Trap staining showed that Mitf overexpression increased osteoclast differentiation and mitigated osteoclastogenesis’s inhibition by Mul-A (Fig. 5A–C). A similar effect was observed in the bone resorption assay (Fig. 5D-E). The impact of Mitf on genes specific to osteoclasts was investigated. Compared to the GFP group, the transcription of Nfatc1, Trap, Ctsk, Atp6v0d2, Oscar, and Mmp9 was upregulated in the Mitf overexpression group. Repression of these genes by Mul-A was attenuated by Mitf overexpression (Supplementary Fig. 5A–F). The changes in the protein levels of Nfatc1, Ctsk, Trap, and c-Fos were consistent with the above results (Fig. 5F–J). These data indicate that Mitf is an enhancer of osteoclastogenesis and that Mul-A inhibits the osteoclast’s maturation through Mitf.

### Autophagy inducers Rapamycin and Torin 1 rescue the inhibitory effects of Mul-A on osteoclasts

**Fig. 6:**
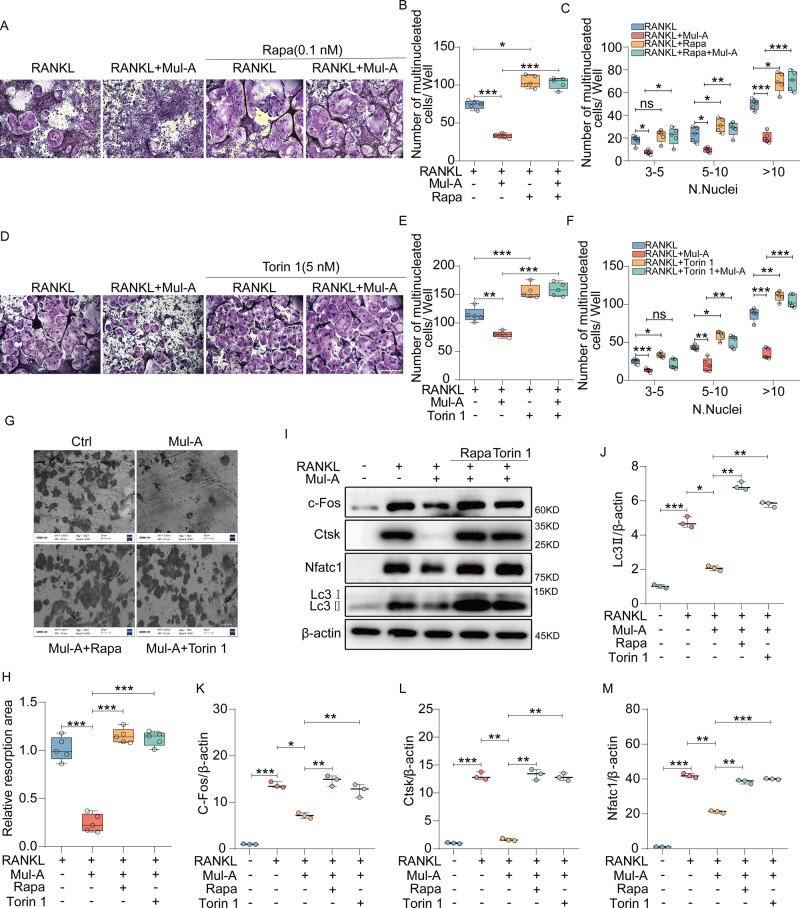
Autophagy inducers Rapamycin and Torin 1 rescue the inhibitory effects of Mul-A on osteoclasts. **A** BMMs were treated with 30 ng/mL M-CSF, 50 ng/mL RANKL, and Rapamycin (Rapa) (0.1 nM) in the presence or absence of Mul-A for 6 days. The cells were then fixed in 4% paraformaldehyde for 15 min and stained with Trap (*n* = 5). Scale bar = 200 μm. **B**, **C** Quantitative analysis of the number of Trap-positive multinuclear cells per well (*n* = 5). **D** BMMs were treated with 30 ng/mL M-CSF, 50 ng/mL RANKL, and Torin 1 (5 nM) in the presence or absence of Mul-A for 6 days. Then the cells were fixed in 4% paraformaldehyde for 15 min and stained with Trap (*n* = 5). Scale bar = 200 μm. **E**, **F** Quantitative analysis of the number of Trap-positive multinuclear cells per well (*n* = 5). **G** BMMs were incubated with 30 ng/mL M-CSF and 50 ng/mL RANKL for 6 days. After the formation of mature osteoclasts, the cells were treated with or without Rapa (0.1 nM), Torin 1 (5 nM), and Mul-A for 4-5 days continuously. Scanning electron microscopy was utilized to observe the number and area of absorption pits (*n* = 5). Scale bar = 50 μm. **H** Quantitative analysis was conducted to determine the area of bone resorption pits (*n* = 5). **I** BMMs were treated with 30 ng/mL M-CSF, 50 ng/mL RANKL, Rapamycin (0.1 nM), and Torin 1 (5 nM) in the presence or absence of Mul-A for 6 days to extract cell lysates. Western blot analysis was performed to assess the expression of Nfatc1, c-Fos, Ctsk, and Lc3 (*n* = 3). **J**–**M** Quantitative analysis was conducted to determine the relative grayscale intensity of the protein bands (*n* = 3). The control group was added with an equivalent DMSO. Data were presented as the median and interquartile range (IQR). *P < 0.05, **P < 0.01, ***P < 0.001.

Considering the role of Mul-A in autophagy modulation during osteoclastogenesis, Rapamycin and Torin 1, two widely used autophagy agonizts, were applied [41–44]. Rapamycin and Torin 1’s effects on osteoclast differentiation were determined. As shown, both Rapamycin and Torin 1 promoted osteoclastogenesis at slight concentrations, and the optimum concentrations were 0.1 nM and 5 nM, respectively, which indicated that moderate activation of autophagy is positive for osteoclastogenesis (Supplementary Fig. 6A, B). Rapamycin and Torin 1 were then applied to BMMs along with Mul-A during osteoclastogenesis to investigate whether they could rescue the anti-osteoclastogenic effects of Mul-A. We found that treatment with Rapamycin and Torin 1 accelerated the osteoclasts’ maturation and improved their bone absorption capacity using Trap staining and bone resorption assay. Both reagents were able to effectively rescue the inhibitory effects of Mul-A on osteoclastogenesis and osteoclast function (Fig. 6A–H). The expression of osteoclast-specific proteins was determined by western blotting. Rapamycin and Torin 1 promoted the expression of Ctsk, c-Fos, and Nfatc1 while mitigating the suppression caused by Mul-A. Importantly, Mitf’s expression was not influenced by Rapamycin and Torin 1. (Fig. 6I–M, Supplementary Fig. 7). Hence, Mul-A exerts its inhibitory role in osteoclastogenesis through the regulation of autophagy.

### Mul-A ameliorates the bone loss induced by ovariectomy

**Fig. 7:**
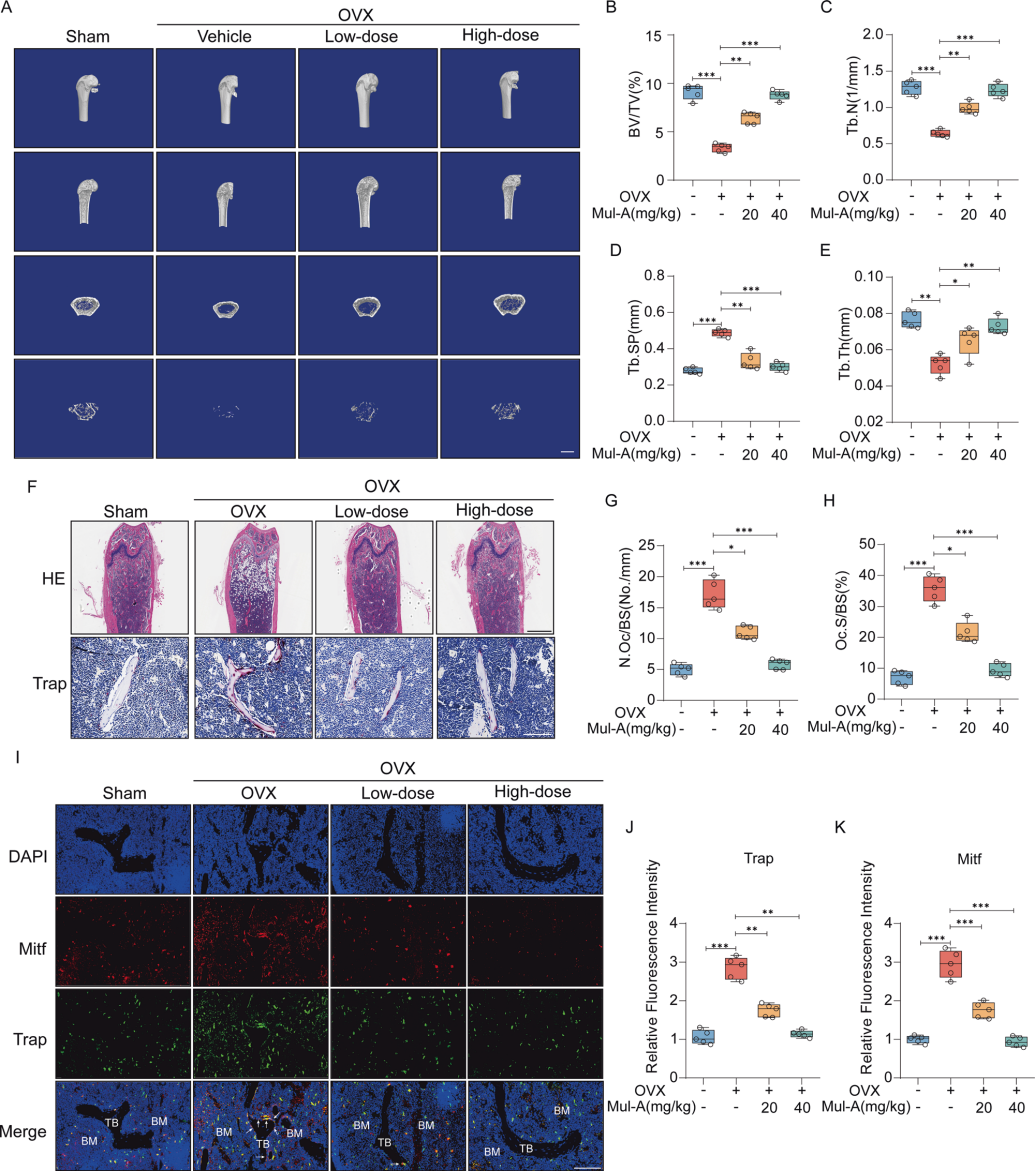
Mul-A ameliorates the bone loss induced by ovariectomy. **A** Micro-CT was utilized to reconstruct the trabecular structure of the distal femur in mice (*n* = 5). Scale bar = 1 mm. **B**–**E** CT-analysis was conducted to calculate various distal femoral microstructure parameters, including bone volume/tissue volume (BV/TV), trabecular number (Tb. N), trabecular thickness (Tb. Th), and trabecular separation (Tb. Sp) (*n* = 5). **F** Histological examination with HE staining and Trap staining was performed on mouse distal femur samples from different groups (*n* = 5). Scale bar = 1 mm, Scale bar = 100 μm. **G, H** Quantitative analysis was carried out to determine the number of osteoclasts per bone surface (N.Oc/BS) and osteoclast surface per bone surface (Oc.S/BS) (*n* = 5). **I** An immunofluorescence co-localization assay was conducted to assess the expression levels of Trap and Mitf in the distal femur (*n* = 5). Scale bar = 100 μm. **J**, **K** Quantitative analysis of the fluorescence intensity of Trap and Mitf (*n* = 5). The Sham group and OVX group were administered a placebo consisting of 10% DMSO and 90% corn oil. Data were presented as the median and interquartile range (IQR). *P < 0.05, **P < 0.01, ***P < 0.001.

An ovariectomy (OVX) model was constructed to explore the curative effects of Mul-A on osteoporosis in vivo. Compared to the sham group, micro-CT images demonstrated that mice in the OVX+vehicle group exhibited a dramatic reduction in bone mass. In contrast, Mul-A administration counteracted estrogen deficiency-associated osteoporosis in a concentration-dependent manner (Fig. 7A). Quantitative analysis revealed that BV/TV, Tb. N, and Tb. Th declined in the OVX+vehicle group, which was reversed by Mul-A. Tb. Sp and BS/BV showed the opposite (Fig. 7B–E, Supplementary Fig. 8A). H&E and Trap staining indicated that compared with the sham group, the bone trabeculae were noticeably decreased in the OVX+vehicle group, accompanied by an increase in osteoclasts surrounding the trabeculae bone. However, Mul-A treatment ameliorated these destructions induced by OVX (Fig. 7F–H). Considering the inhibitory role of Mul-A on Mitf in vitro, an immunofluorescence assay was conducted. The results showed that ovariectomy increased the expression of Trap and Mitf in the mouse distal femur, whereas Mul-A significantly curbed it (Fig. 7I–K). In addition, the vital organs of mice from each group were preserved, and H&E staining indicated that high-dose Mul-A (40 mg/kg) exerted no obvious toxicity on mice organs (Supplementary Fig. 8B). In summary, Mul-A shows a therapeutic potential against osteoporosis and inhibits Mitf expression in vivo.

### Mul-A exerts no obvious impact on osteogenesis

Bone formation, another important biological process that maintains homeostasis of bone metabolism, is mainly modulated by osteoblasts. Therefore, it is important to test the effects of Mul-A on osteogenesis. The cell viability assay (CCK8) results showed that osteoblast activity was not decreased by treatment with Mul-A for 48 or 96 h (Supplementary Fig. 9A, B). Accordingly, pre-osteoblasts isolated from skulls of suckling mice were cultured in an OB medium along with different dosages of Mul-A (0, 20, 40, 80 μM) to differentiate into mature osteoblasts. Alkaline phosphatase (ALP) staining and Alizarin Red S(ARS) staining assays were performed at 10 and 21 days, respectively. The results clarified that neither the activity of alkaline phosphatase nor the formation of calcium nodules was altered in the presence of Mul-A (Supplementary Fig. 9C, D). Similarly, qRT-PCR data showed that Mul-A had no obvious influence on the transcription of osteoblast-specific genes during OB differentiation (Supplementary Fig. 9E–H). Moreover, a calcein-labeling assay was used to verify the effect of Mul-A on osteogenesis in vivo. Quantitative analysis indicated that the mineralization apposition rate (MAR) was similar between the three groups (OVX + Vehicle, OVX + Low-dose, and OVX + High-dose) (Supplementary Fig. 9I–J). These findings suggest that Mul-A had no obvious impact on osteogenesis.

## Discussion

The pathogenesis of osteoporosis is complex; therefore, clarifying its mechanism and finding effective management measures are the concentrations of current research. In this study, we linked autophagy to osteoporosis. Taking Mitf as the target of Mul-A in regulating autophagy, the pathological mechanism by which Mul-A inhibits osteoclast formation and improves osteoporosis caused by estrogen deficiency was elucidated.

Autophagy, a highly evolutionarily conserved process of cellular catabolism, is a self-protective mechanism produced by cells in response to various internal and external stress factors [45, 46]. Recently, the contribution of autophagy in osteoporosis progression has gained attention. Although multiple pharmacological effects of Mul-A have been well studied, the role of Mul-A in autophagy has not yet been reported. In our study, we found that increased Lc3II expression is accompanied by P62 degradation during osteoclast formation. These findings show that the level of autophagy gradually increases with RANKL stimulation. Moreover, we demonstrated that Mul-A inhibited autophagic flux. The expression of autophagy-related proteins, such as Lc3, Atg16l1, and Atg5, dramatically decreased after Mul-A treatment. Adversely, the degradation of P62 was blocked by Mul-A. Transmission electron microscopy (TEM) and Confocal Laser Scanning Microscope (CLSM) results indicated that Mul-A suppressed the formation of autophagosomes and the fusion of autophagosomes and lysosomes.

Mitf was introduced to clarify the mechanism by which Mul-A regulates autophagy. Mitf involves autophagy regulation and is restrained by Mul-A in melanocytes [47]. In this study, Mul-A down-regulated Mitf expression in osteoclasts and prevented the nucleocytoplasmic shuttle of Mitf induced by RANKL. In addition, the role of Mitf in autophagy regulation by Mul-A was explored. Overexpression of Mitf significantly promoted autophagy. Importantly, the excessive expression of Mitf partly rescued the inhibition of autophagy by Mul-A. Accordingly, we conclude that the Mitf partly mediates the adverse effects of Mul-A on autophagy. Similarly, Mitf overexpression significantly increased the number of Trap-positive multinuclear osteoclasts, area of bone resorption pits, and expression of osteoclast-related genes. Interestingly, Mul-A’s inhibitory effect on osteoclast formation decreased after Mitf overexpression. Finally, we demonstrated that Rapamycin and Torin 1 overthrow the inhibitory effect of Mul-A on osteoclast differentiation and absorption, and similar results were observed for osteoclast marker proteins. Accumulating data indicate that Mul-A suppresses autophagy by regulating Mitf, subsequently reducing osteoclastogenesis.

Although we demonstrated that Mul-A suppressed Mitf’s expression and nuclear translocation, the underlying mechanism was not studied. Epigenetic modifications regulate Mitf expression. Lauss M. et al. [48] confirmed that the transcription of Mitf was negatively correlated with the methylation of CpG islands at the transcription start site (TSS). Moreover, P300 and CREB-binding protein (CBP), major enzymes that catalyze the acetylation of histone H3-lysine 27(H3K27), bind to the activation domain of Mitf, resulting in its transcriptional activation [49]. Whether Mul-A inhibits Mitf expression by regulating epigenetic modifications requires further study. Interestingly, activated Mitf increases the pH in intracellular. An alkaline pH environment activates CBP/P300, stimulating H3K27 acetylation in the Mitf downstream gene promoter. This results in increased expression of Mitf downstream genes [50]. Whether this positive feedback exists in the regulation of autophagy and osteoclast formation by Mitf requires further exploration. Similarly, the mechanism underlying the nuclear localization of Mitf has not yet been elucidated. Activated mTOR phosphorylates Mitf, allowing it to bind to YWHA/14-3-3 and remain in the cytoplasm. Dephosphorylated Mitf is transferred to the nucleus to activate autophagy and lysosomal biosynthesis when mTOR is depleted [51]. Nevertheless, our results showed that RANKL promoted the nuclear transfer of Mitf. Therefore, we speculate that RANKL may promote Mitf’s dephosphorylation by regulating mTOR’s activity, which warrants further research.

**Fig. 8:**
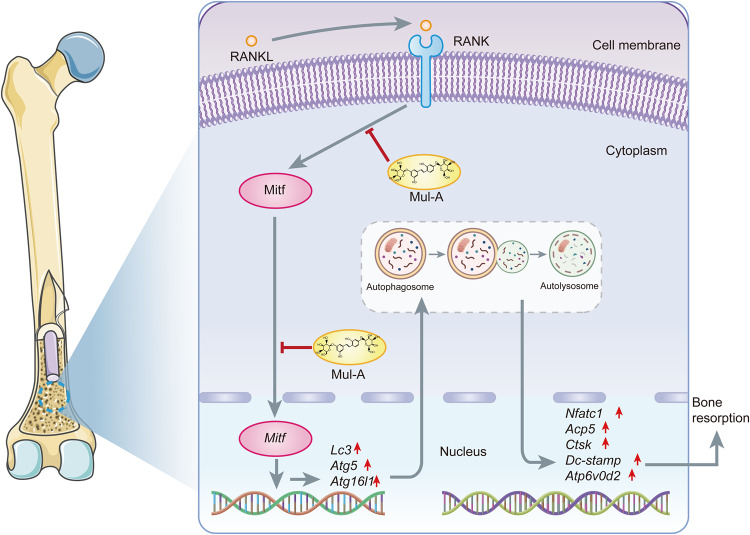
A schematic diagram showing the mechanism of Mul-A inhibiting osteoclast differentiation. Mul-A inhibited autophagy by downregulating Mitf expression and nuclear transfer, subsequently impairing osteoclast differentiation and function.

In general, we found that Mul-A suppressed osteoclastogenesis and function in vitro without affecting osteogenesis. In vivo, Mul-A ameliorated bone loss caused by estrogen deficiency without causing toxicity to mice’s organs. Mechanistically, Mul-A inhibited autophagy by downregulating Mitf expression and nuclear transfer, subsequently impairing osteoclast differentiation and function (Fig. 8). Accordingly, the Mitf-autophagy axis may be a new pathway by which Mitf regulates osteoclastogenesis. Mul-A has the potential to treat bone metabolic disorders like osteoporosis.

## Materials and methods

### Reagents

Mulberroside A (Cat#HY-N0619), Rapamycin (Cat# HY-10219), and Torin 1 (Cat# HY-13003) were obtained from MedChemExpress (New Jersey, USA), dissolved in DMSO, and then stored at −20 °C. Fetal bovine serum (Cat#10099141C), alpha-Minimum Essential Medium (Cat#12571063), and penicillin/streptomycin (Cat#15410163) were purchased from Gibco (New York, USA). Recombinant soluble mouse M-CSF (Cat# 416-ML-010/CF) and RANKL (Cat# 462-TEC-010/CF) were purchased from R&D (Minnesota, USA). Cell Counting Kit-8 (CCK-8) (Cat# K1018) was purchased from APExBIO Technology (Houston, USA). Tartrate-resistant acid phosphatase (Trap) staining dye (Cat# 3863, Cat# 3871, Cat# 914, Cat# 3872) was obtained from Sigma-Aldrich (Missouri, USA). DAPI (Cat# 40728ES03) and Hieff^®^ qPCR SYBR Green Master Mix (Cat# 11201ES03) were obtained from Yeasen (Shanghai, China). Phalloidin (Cat# CA1640) and 2.5% glutaraldehyde (Cat#P1126) were purchased from Solarbio (Beijing, China). 5XEvo M-MLV RT Master Mix (Cat#AG11603) was derived from Accurate Biology (Hunan, China). The Enhanced BCA Protein Assay Kit (Cat#P0009) and Triton X-100 (Cat# ST797) were supplied by Beyotime Biology (Shanghai, China). Osmic acid, Spurr embedding agent, and uranyl acetate were obtained from SPI-CHEM (Pennsylvania, USA). Mitf-Adenovirus and mRFP-GFP-Lc3 (Cat# HB-AP210000) adenovirus were purchased from Hanbio (Shanghai, China). BCIP/NBT Kit (Cat# CW0051S) was obtained from CWBIO (Jiangsu, China) and Alizarin Red S (Cat# IA5140) was purchased from Solarbio. Raw264.7 cells (Cat# TCM13, RRID: CVCL_0493) were derived from the National Collection of Authenticated Cell Cultures (Shanghai, China). Supplementary Table. 1 shows all antibodies used herein.

### Cytotoxicity assay

The toxicity of Mul-A on the proliferation activity of bone marrow-derived mononuclear macrophages (BMMs) was assessed employing the CCK8 kit. BMMs were seeded at a density of 10^4 cells per well in 96-well plates and cultured with 30 ng/mL M-CSF and various concentrations of Mul-A for 48 or 96 h. Subsequently, the cells were incubated in a serum-free medium containing 10% CCK8 buffer at 37 °C for 2 h. The absorbance at 450 nm was detected utilizing an ELX800 absorbance microplate reader (BioTek Instruments, Winooski, VT, USA) to evaluate the toxicity of Mul-A on the BMMs.

### Osteoclastogenesis in vitro and F-actin ring staining assay

The BMMs for differentiation were obtained from the femur and tibia bone marrow of 7-week-old male C57BL/6 mice. The cells were cultured in α-MEM medium supplemented with 10% FBS and 1% penicillin/streptomycin in a 10 cm cell culture dish. Additionally, 30 ng/mL M-CSF was applied to facilitate proliferation. The medium was refreshed every two days. Upon reaching a cell density of 80–90%, the cells were trypsinized and planted into 96-well plates at a density of 8 × 10^3 cells per well. After 24 h, the medium was replaced with fresh medium containing 30 ng/mL M-CSF and 50 ng/mL RANKL for 5-7 days. The medium was changed every two days until BMMs differentiated into multinucleated osteoclasts. The culture medium was then removed, and the cells were fixed with 4% paraformaldehyde for 15 min. Subsequently, the cells were washed thrice with PBS and incubated with 100 μL of Trap staining buffer per well at 37 °C for 30–60 min. The number of Trap^+^ multinuclear cells was counted using a microscope.

BMMs were exposed to varying concentrations (0, 20, 40, and 80 µM) of Mul-A during the 5-7 day differentiation period. Once mature osteoclasts were formed in the control group, they were fixed with 4% paraformaldehyde for 15 min and washed with PBS. Cell membrane permeabilization was achieved by treating the cells with Triton X-100 for 30 min. The F-actin cytoskeleton was stained with phalloidin for 30–90 min, while the nucleus was counterstained with DAPI. Subsequently, the fluorescence microscope was used to visualize the length of F-actin, and the quantification was performed using ImageJ software.

### Bone resorption assay

After sterilization, bovine bone slices were carefully placed in a 96-well plate. BMMs were then seeded onto the bovine bone slices with an initial cell count of 8 × 10^3 cells per well. The cells were subsequently stimulated with 30 ng/mL M-CSF and 50 ng/mL RANKL for 5–7 days to induce mature osteoclast formation. After mature osteoclasts were formed, different concentrations of Mul-A (0, 20, 40, and 80 µM) were added, followed by an additional incubation period of 4-5 days. After completion of the treatment, the bovine bone slices were carefully removed and subjected to observation of bone resorption using a scanning electron microscope (ZEISS GEMINI-300). The extent of bone resorption was quantified by measuring the area of bone resorption pits using ImageJ software.

### Quantitative real-time PCR (qRT-PCR)

mRNA was extracted from the treated cells using the mRNA extraction kit (AG, Hunan, China). The concentration of mRNA was then determined using the Nano-Drop2000 spectrophotometer. Subsequently, cDNA was synthesized using reverse transcription reagents from AG. For the qRT-PCR reaction, the following components were employed: 5 µL of SYBR Green qPCR Master Mix, 3 µL of distilled water, 1 µL of cDNA, and 0.5 µL each of the forward and reverse primers. The ABI Prism 7500 System (ABI, Foster City, CA, USA) was employed to quantify the expression level of the target genes. To normalize the cDNA amounts, β-Actin was used as a reference gene. Supplementary Table. 2 lists the primer sequences of the target genes and reference genes.

### Western blot assay

The cells were effectively lysed at a temperature of 4 °C or on ice for 30 min using RIPA lysate (Beyotime Cat# P0013E) added with protease inhibitor PMSF (Beyotime Cat# ST506). Following this, the lysate was centrifuged at 12,000 rpm for 10 min at 4 °C. The resulting precipitate was discarded, and the remaining liquid, known as the total protein extract, was collected. To accurately measure the protein quantities, the BCA kit was employed. Next, the extracted protein was separated using electrophoresis through a 10% SDS-PAGE gel and transferred onto a PVDF membrane (BIO-RAD Cat# 1620256). To prevent any nonspecific binding, the membrane was sealed with 5% skimmed milk (Fdbio Science Cat# FD0080) for approximately 30–60 min at a temperature of 25 °C. Specific primary antibodies were then incubated with the PVDF membrane overnight at 4 °C to enable the detection of target proteins. Following primary antibody incubation, the membrane was washed with TBST three times and incubated with HRP-conjugated secondary antibodies (1:5000, goat anti-mouse IgG, Cat# FDM007; goat anti-rabbit IgG, Cat# FDR007, Fude Biological Technology Co., Ltd, Hangzhou, China) for 60 min at ambient temperature. The secondary antibodies were diluted in TBST (Beyotime Cat# ST673) and used once only. After three additional washes with TBST, the membrane was visualized using the Amersham Imager 600 (GE, USA). Quantitative analysis of the protein bands was performed using ImageJ in a blinded manner.

### Transmission electron microscopy

The pre-osteoclasts were detached and subjected to centrifugation at 1000 rpm for 3 min. The obtained cell pellet was collected and subjected to overnight fixation using 2.5% glutaraldehyde. Next, the cells were thoroughly rinsed with PBS three times. To prevent any potential damage from subsequent centrifugation steps, the cells were embedded in agarose. Following embedding, the samples were fixed for 2 h in a 1% osmic acid solution and rinsed again with PBS. To facilitate dehydration, the samples were treated with progressively increasing proportions of ethanol (30%, 50%, 70%, and 80%) for 15 min each. This was followed by treatment with 90% and 95% acetone for 15 min, respectively. To complete the dehydration process, the samples were subjected to two rounds of treatment with 100% acetone for 20 min each.

In the subsequent infiltration stage, the cells were embedded using a mixture of Spurr embedding agent and acetone in different proportions. The first mixture was applied for 1 h, while the second mixture was applied for 3 h. Finally, the samples were infiltrated with pure Spurr embedding agent overnight. Afterward, the samples were heated at 70 °C overnight and then sectioned into ultrathin slices measuring 70–90 nm using an ultratome (LEICA EM UC7). The slices were treated with uranyl acetate and alkaline lead citrate for 10 min. The ultrastructure of the cells was observed using a transmission electron microscope (Hitachi H-7650).

### Autophagic flux assay

BMMs were inoculated at a density of 1 × 10^4 cells per well in 96-well plates. After 24 h, The cells were infected with mRFP-GFP-Lc3 adenovirus to determine the optimal multiplicity of infection (MOI). BMMs were subsequently plated in 24-well plates at a density of 1.5 × 10^5 cells per well and transfected with the previously determined optimal MOI for 24 h. The growth medium was substituted, and the cells were then placed in a 5% CO_2_ incubator at 37 °C for an additional 24 h. Afterward, the cells were collected and plated at a density of 5 × 10^4 cells per well in confocal culture dishes. Once fully adhered, the cells were incubated with 30 ng/mL M-CSF, 50 ng/mL RANKL, and 80 µM Mul-A for four days. Following three times PBS washes, the autophagy flux was examined using a laser-scanning confocal microscope (Nikon).

It is worth noting that GFP fluorescence is highly sensitive to changes in pH, rendering it non-detectable under acidic conditions. Thus, the presence of only red fluorescence signifies a fusion between autophagosomes and lysosomes in the late stages of autophagy. On the other hand, yellow puncta (representing fused GFP and RFP signals) indicate the presence of early autophagosomes.

### Cell transfection in vitro

Transfection of 3×Flag-Mitf adenovirus (Hanbio) was conducted following the provided protocols. The cells were infected with the adenovirus at the optimal concentration and incubated for 24 h. Subsequently, the culture medium was changed, and the cells were continuously cultured for an additional 24–48 h. To assess the transfection efficiency, both qRT-PCR and western blotting were employed.

### Osteoblastogenesis assay in vitro

Osteoblasts were extracted from the skulls of 3-day-old male suckling mice. The osteogenic medium used for culturing the osteoblasts consisted of DMEM supplemented with 100 µM vitamin C and 10 mM β-glycerol. The osteoblasts were subjected to treatment with different concentrations of Mul-A (0, 20, 40, 80 µM) for seven days. After the treatment, the cells were rinsed thrice with PBS and fixed with 4% paraformaldehyde for 20 min. Subsequently, the Alp staining solution (CWBIO) was utilized to stain the alkaline phosphatase. In another experiment, osteoblasts were induced using the same protocol for 21 days. After the induction, the cells were immobilized using 4% paraformaldehyde and stained with 0.5% Alizarin Red S (Solarbio) at room temperature for 10 min to visualize the mineralized matrix. Images of the different experimental groups were captured using an optical scanner.

### Animal model of bilateral ovariectomy

All operations on animals were authorized by the ethics committee of Sir Run Run Shaw Hospital (Zhejiang University affiliated, Hangzhou, Zhejiang). All experiments followed the Ethical Conduct in the Care and Use of Animals guideline developed by the Council for International Organizations of Medical Sciences. All the mice used in the experiment were purchased from Ziyuan Experimental Animal Technology Co., Ltd (Hangzhou, China).

The mice were accommodated in a controlled barrier facility, maintained under pathogen-free conditions with regulated temperature and humidity. A 12-h light/dark cycle was implemented. The mice were provided unrestricted access to both food and water. Using a double-blind method, the 20 12-week-old female mice were randomly allocated into four groups: Sham, OVX+Vehicle, OVX+Low-dose (20 mg/kg), and OVX+High-dose (40 mg/kg). Before the surgery, all surgical instruments were sterilized at high temperatures, and the surgical incision was thoroughly disinfected with iodophor. The mice were then anesthetized with 4% chloral hydrate (0.1 ml/kg body weight). Proper measures were taken to monitor and maintain the mice’s temperature and respiration during the procedure. Bilateral ovariectomy was performed on the OVX+Vehicle, OVX+Low-dose, and OVX+High-dose groups, while the Sham group underwent sham surgery. After the surgery, the wounds of the mice were disinfected using iodophor. Any mice exhibiting signs of wound inflammation that severely affected their vital signs were excluded from the study. Two weeks after the surgery, the OVX+Low-dose and OVX+High-dose groups received intraperitoneal injections of Mul-A at doses of 20 mg/kg or 40 mg/kg, respectively, every two days. The Sham group and OVX group were administered with a placebo consisting of 10% DMSO and 90% corn oil. After six weeks, all the mice were euthanized using isoflurane anesthesia. Bilateral femurs and visceral organs were collected and fixed with 4% paraformaldehyde for 48 h. Micro-CT scanning analysis was performed on the right femur. Further experiments on the decalcified femur included immunohistochemistry, immunofluorescence, H&E staining, and Trap staining. The organs were also stained with H&E to assess the toxicity of Mul-A on mice’s viscera.

Despite being a commonly used animal model for studying osteoporosis, this model does have limitations. The bilateral ovariectomy does not fully mimic the clinical pathogenesis of estrogen deficiency-induced osteoporosis. In osteoporosis patients, although ovarian function declines, the ovarian interstitial cells still retain some endocrine function.

### Micro-CT scanning

After 48 h of fixation, the femur was subjected to high-resolution micro-CT scanning using the SkyScan 1275 system (Bruker microCT, Kontich, Belgium). The scanning site was carefully chosen in the distal femur, and the following parameters were employed: an isometric resolution of 9 μm, X-ray energy set at 60 kV, and 60 mA. To visualize the femur in three dimensions, the acquired data was processed and reconstructed using DataViewer software (version 1.5.6.2). Quantitative analysis of various parameters including the bone surface area to bone volume ratio (BS/BV), bone volume to tissue volume ratio (BV/TV), trabecular thickness (Tb. Th), trabecular number (Tb. N), trabecular separation (Tb. SP), and bone mineral density (BMD) were performed using CTAn software (version 1.20.8.0). Additionally, CTvox software (version 3.3) was used to construct three-dimensional reconstruction images of the femur.

### Bone histomorphometry and calcein labeling

After fixation, the femur was decalcified in a 10% EDTA solution for 14 days. Once adequately softened, the femur was encased in paraffin and processed into tissue sections. Subsequently, the tissue sections underwent Trap staining, H&E staining, immunohistochemistry, and immunofluorescence tests following the standard procedures of dewaxing and dehydration. H&E staining was conducted to visually assess the number of trabecular bones, while Trap staining was employed to detect the osteoclasts surrounding the trabecular bones.

For dynamic assessment of osteoblast activity in vivo, a 2.5 mg/ml calcein solution (200 µL each) was injected into the abdominal cavity of the mice during the first ten days and the first three days before euthanasia. The femur from one side was collected and processed into tissue sections, which were subsequently analyzed using a MAGSCANNER KF-PRO-120 (KFBIO) system. The migration distance marked by calcein serves as an indicator of osteoblast activity in vivo.

### Immunofluorescence (IF)

Paraffin sections were deparaffinized and heated at 95–100 °C for 20 min with 0.01 M sodium citrate solution (PH = 6) for antigen repair. Following cooling, the sections were washed three times with PBS. Subsequently, the sections were covered with 0.1% Triton X-100 for 30 min to facilitate permeabilization, followed by blocking with 5% BSA for 60 min to prevent nonspecific binding. For immunostaining, the specific primary antibody against Mitf (dilution 1:200) was applied, ensuring complete coverage of the sections, and incubated overnight at 4 °C. After being washed with PBS, the DyLight 594 AffiniPure Goat Anti-Rabbit IgG (H + L) secondary antibody (dilution 1:200, Cat#: FD0129, Fude Biological Technology Co., Ltd, Hangzhou, China) was applied and combined with primary antibody for 60 min at ambient temperature in the dark (following subsequent steps in the absence of light). Subsequently, the sections were rinsed thrice with PBS and stained with DAPI (diluted 1:1000 in PBS) for 15 min to counterstain cell nuclei. After a thorough cleaning, the fluorescence intensity of the target protein was detected using the MAGSCANNER KF-PRO-120 system. The ImageJ was performed for quantitative analysis.

### Statistical analysis

The aforementioned tests were conducted in triplicate at least to ensure reproducibility. Statistical analysis was conducted utilizing Prism 9 software (GraphPad Software, Inc., San Diego, CA, USA). The data were graphically presented as boxplots, depicting the median and interquartile ranges (IQR) along with individual data points. For comparisons between two groups, the Student’s t-test was utilized, while for multiple-group comparisons, one-way or two-way ANOVA followed by Tukey’s post hoc analysis was employed as deemed appropriate. *P* < 0.05 was considered statistically significant. *P < 0.05, **P < 0.01, or ***P < 0.001.

### Supplementary information

Supplementary materials

## Data Availability

The data that support the findings of this study are available from the corresponding author upon reasonable request.
