# Publisher Correction: Restrained Mitf-associated autophagy by Mulberroside A ameliorates osteoclastogenesis and counteracts OVX-Induced osteoporosis in mice

**DOI:** 10.1038/s41420-024-01928-1

**Published:** 2024-07-09

**Authors:** Hong Xue, Zhenhua Feng, Putao Yuan, Li Qiao, Qiliang Lou, Xiangde Zhao, Qingliang Ma, Shiyu Wang, Yang Shen, Huali Ye, Jiao Cheng, Jiying Wang, Shuanglin Wan, Boya Zhang, Peihua Shi, Xuewu Sun

**Affiliations:** 1 Department of Orthopaedic Surgery, Sir Run Run Shaw Hospital, Zhejiang University School of Medicine, Hangzhou, China; 2 Key Laboratory of Musculoskeletal System Degeneration and Regeneration Translational Research of Zhejiang Province, Hangzhou, China; 3 Department of Dermatology, Sir Run Run Shaw Hospital, Zhejiang University School of Medicine, Hangzhou, China., Hangzhou, China

**Keywords:** Macroautophagy, Osteoporosis

Correction to: *Cell Death Discovery* 10.1038/s41420-024-01847-1, published online 15 February 2024

In this artile the equal contribution is missing. This has been omitted during the production process.

It should be read:

Hong Xue, Zhenhua Feng and Putao Yuan contributed equally to this work.

The original article has been corrected.

