# Supplementary material for: Restrained Mitf-associated autophagy by Mulberroside A ameliorates osteoclastogenesis and counteracts OVX-Induced osteoporosis in mice
Source: Cell Death Discov. 2024 Feb 15;10:80. doi: 10.1038/s41420-024-01847-1 (PMC10869803; doi:10.1038/s41420-024-01847-1)
Supplement: Supplementary file 1 — Supplementary materials [file 41420_2024_1847_MOESM1_ESM.pdf]

Supplementary figures

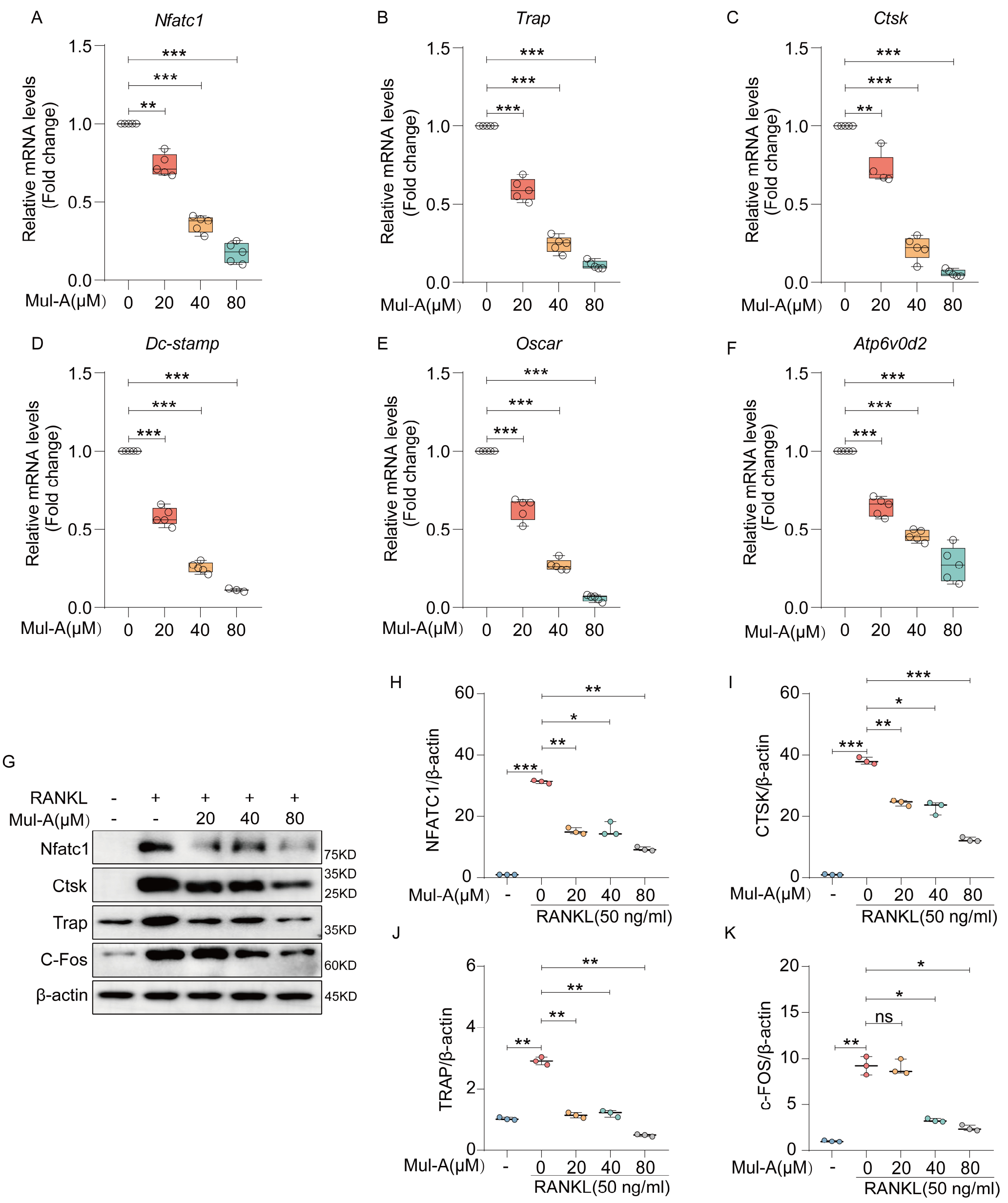

Supplementary Fig.1 Mul-A suppresses the specific gene expression of osteoclast in a dose-dependent manner.

(A-F) BMMs were treated with different concentrations of Mul-A (0, 20, 40, and 80 μM) in the presence of 30 ng/mL M-CSF and 50 ng/mL RANKL for 6 days. The mRNA levels of the aforementioned genes were quantitatively analyzed using quantitative real-time PCR (n = 5). (G) The cells were cultured with Mul-A at concentrations of 0, 20,

40, and 80 μM, along with 30 ng/mL M-CSF and 50 ng/mL RANKL, for 6 days. Total protein was extracted for western blot analysis to assess the changes in Nfatc1, Ctsk, C-Fos, and Trap protein levels (n = 3). (H-K) Quantitative analysis was performed to determine the relative grayscale intensity of the protein bands (n = 3). The control group was added with an equivalent DMSO. Data were presented as the median and interquartile range (IQR). *P<0.05, **P<0.01, ***P<0.001.

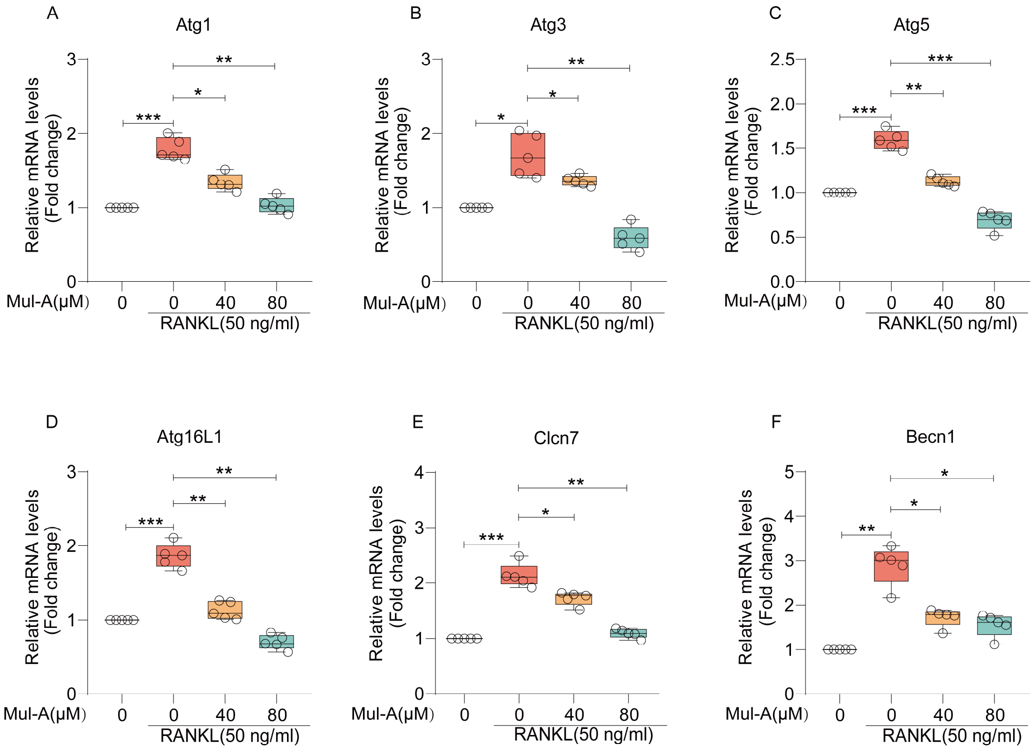

Supplementary Fig. 2 Mul-A down-regulates the activation of autophagy-related genes.

(A-F) BMMs were stimulated with Mul-A (0, 40, 80 μM) and 50 ng/mL RANKL for 3 days. RNA was extracted for Quantitative real-time PCR to analyze the autophagy-related gene expression (n = 5). The control group was added with an equivalent DMSO.

Data were presented as the median and interquartile range (IQR). *P<0.05, **P<0.01, ***P<0.001.

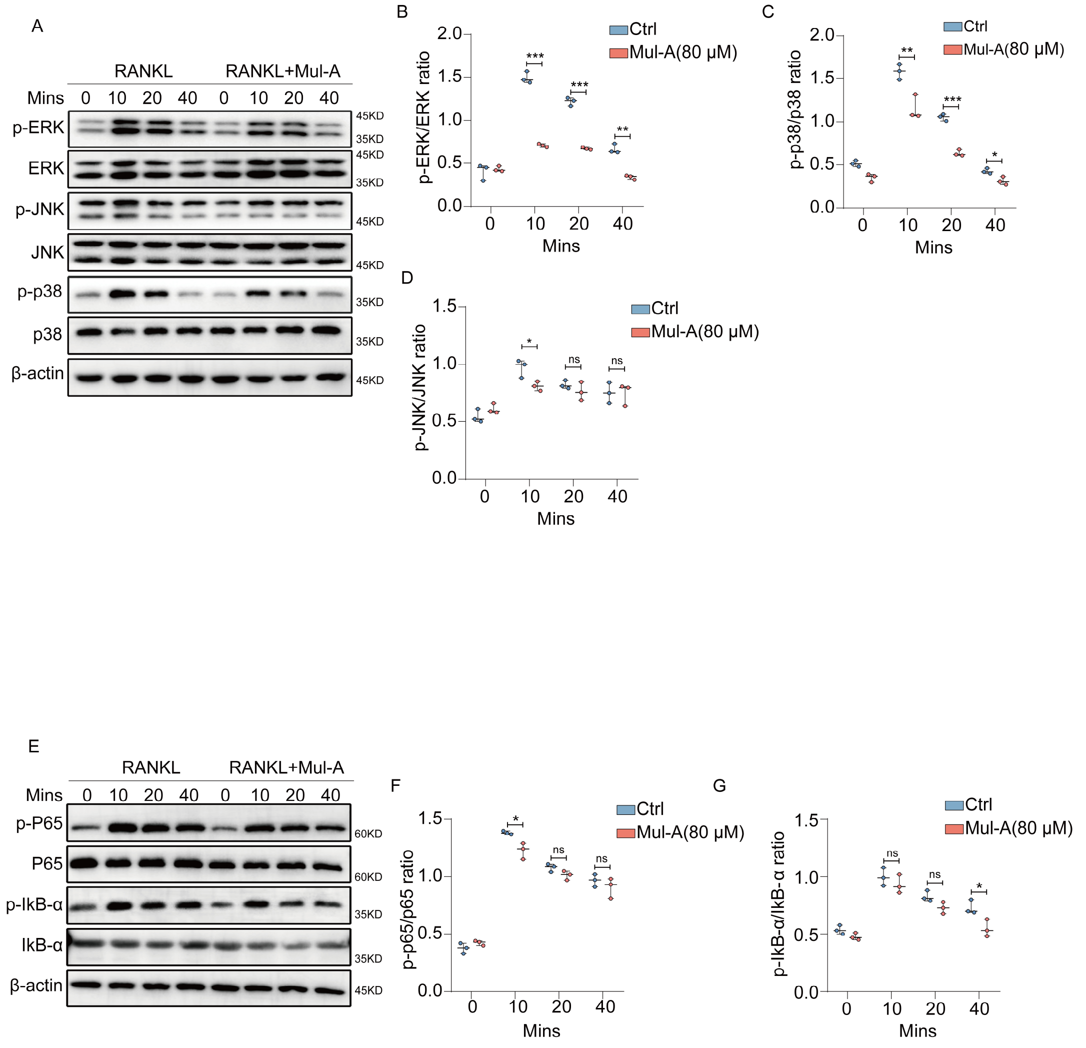

Supplementary Fig. 3 The role of Mul-A on the activation of MAPK and NF-κB pathway.

(A, D) After pretreated with Mul-A, Raw 264.7 cells were stimulated with 50 ng/mL RANKL for 0, 10, 20, and 40 min. The phosphorylation of p38, ERK, JNK, P65, and

IKB-α was detected by western blot (n=3). (B-D, F-G) Quantitative analysis was performed to determine the relative grayscale intensity of the protein bands (n = 3). The control group was added with an equivalent DMSO. Data were presented as the median and interquartile range (IQR). *P<0.05, **P<0.01, ***P<0.001.

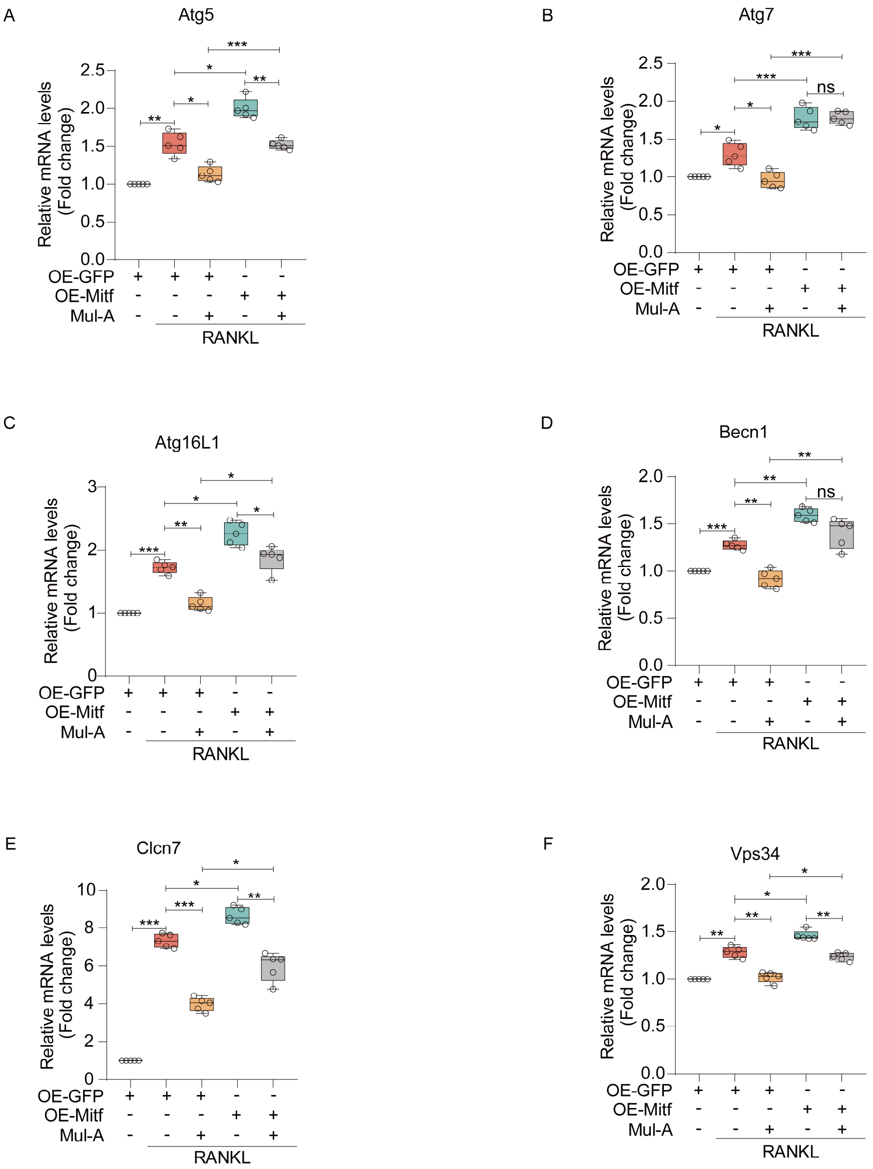

Supplementary Fig. 4 Mitf overexpression rescues the suppresses of Mul-A on autophagy-related genes.

(A-F) BMMs were continuously treated with 30 ng/mL M-CSF and 50 ng/mL RANKL in the presence or absence of Mul-A for 4 days after Mitf transfected. Quantitative real-time PCR was used to analyze the expression of autophagy-related genes (n = 5). The control group was added with an equivalent DMSO. Data were presented as the median and interquartile range (IQR). *P<0.05, **P<0.01, ***P<0.001.

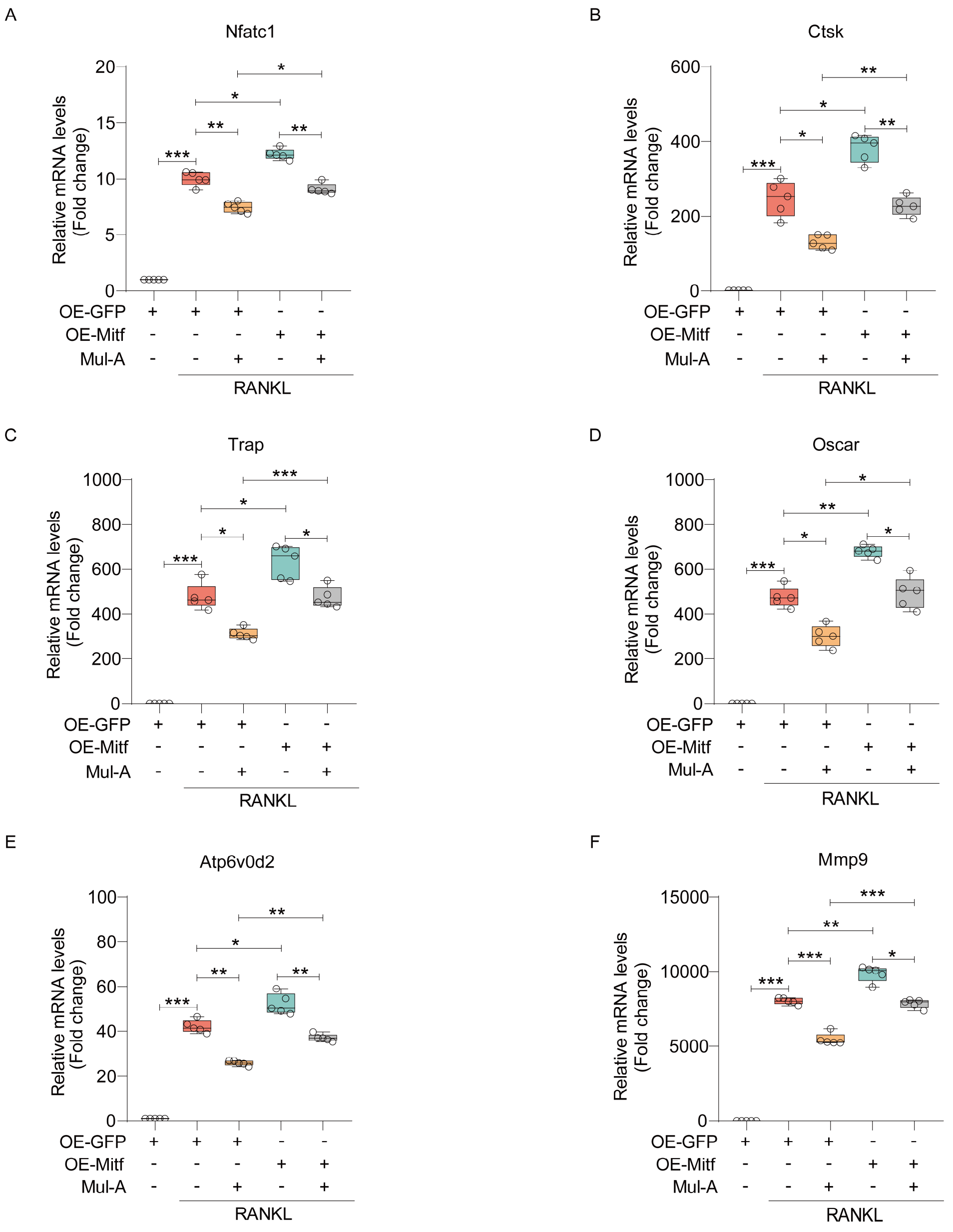

Supplementary Fig. 5 Mitf overexpression attenuates the restraint impact of Mul-A on osteoclast-related genes.

(A-F) After infecting with 3xflag Mitf adenovirus for 2 days, BMMs were treated for 6 days in the conditions of 50 ng/mL RANKL and 80 μM Mul-A. Quantitative real-time PCR was performed to examine the transcriptional activity of osteoclast-related genes (n = 5). The control group was added with an equivalent DMSO. Data were presented as the median and interquartile range (IQR). *P<0.05, **P<0.01, ***P<0.001.

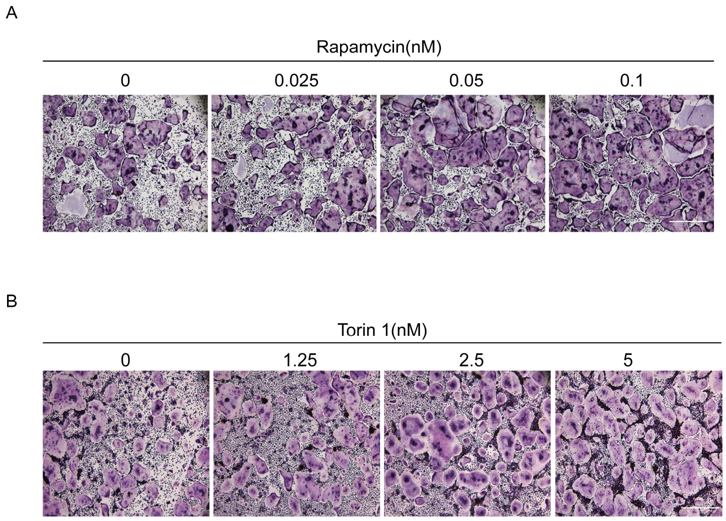

Supplementary Fig. 6 Rapamycin and Torin 1 promote osteoclast differentiation in a certain concentration range.

(A) BMMs were treated with 30 ng/mL M-CSF and 50 ng/mL RANKL along with various concentrations of Rapamycin, for 6 days. The cells were then fixed in 4% paraformaldehyde for 15 min and stained with Trap (n = 5). (B) BMMs were treated

with 30 ng/mL M-CSF and 50 ng/mL RANKL, along with different concentrations of Torin 1, for 6 days. The cells were subsequently fixed in 4% paraformaldehyde for 15 min and stained with Trap (n = 5). Scale bar = 200 μm. The control group was added with an equivalent DMSO. *P<0.05, **P<0.01, ***P<0.001.

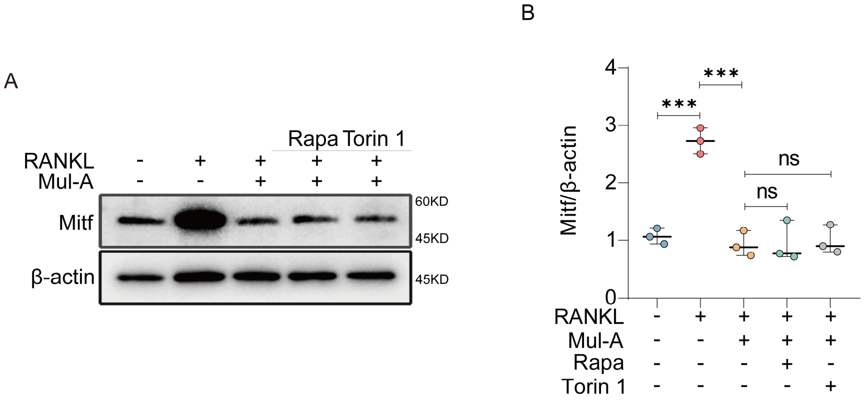

Supplementary Fig. 7 Rapamycin and Torin 1 don't alter the expression level of Mitf.

(A) BMMs were treated with 30 ng/mL M-CSF, 50 ng/mL RANKL, Rapamycin (0.1 nM), and Torin 1 (5 nM) in the presence or absence of Mul-A for 6 days to extract cell lysates. Western blot analysis was performed to assess the expression of Mitf (n = 3). (B) Quantitative analysis was conducted to determine the relative grayscale intensity of

the protein bands (n = 3). The control group was added with an equivalent DMSO. Data were presented as the median and interquartile range (IQR). *P<0.05, **P<0.01, ***P<0.001.

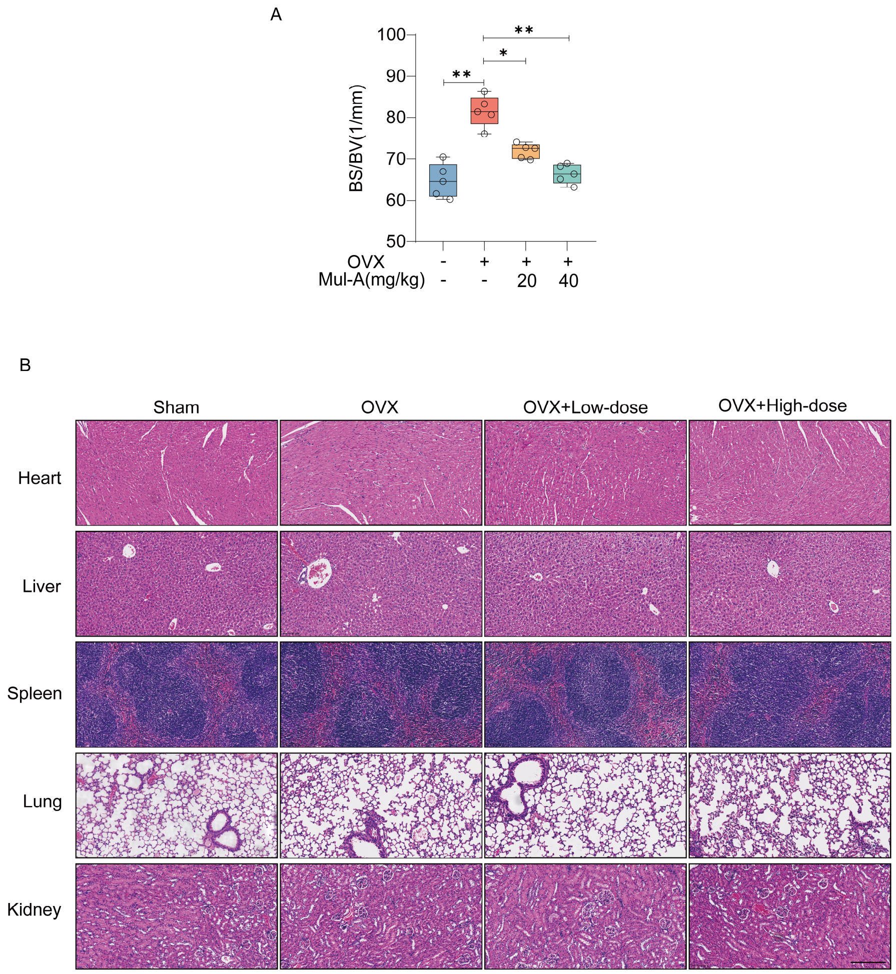

Supplementary Fig. 8 (A) CT-analysis was conducted to calculate bone surface/bone volume (n = 5). (B) HE staining of mouse internal organs (n=5). Scale bar = 200 μm. Data were presented as the median and interquartile range (IQR). *P<0.05, **P<0.01, ***P<0.001.

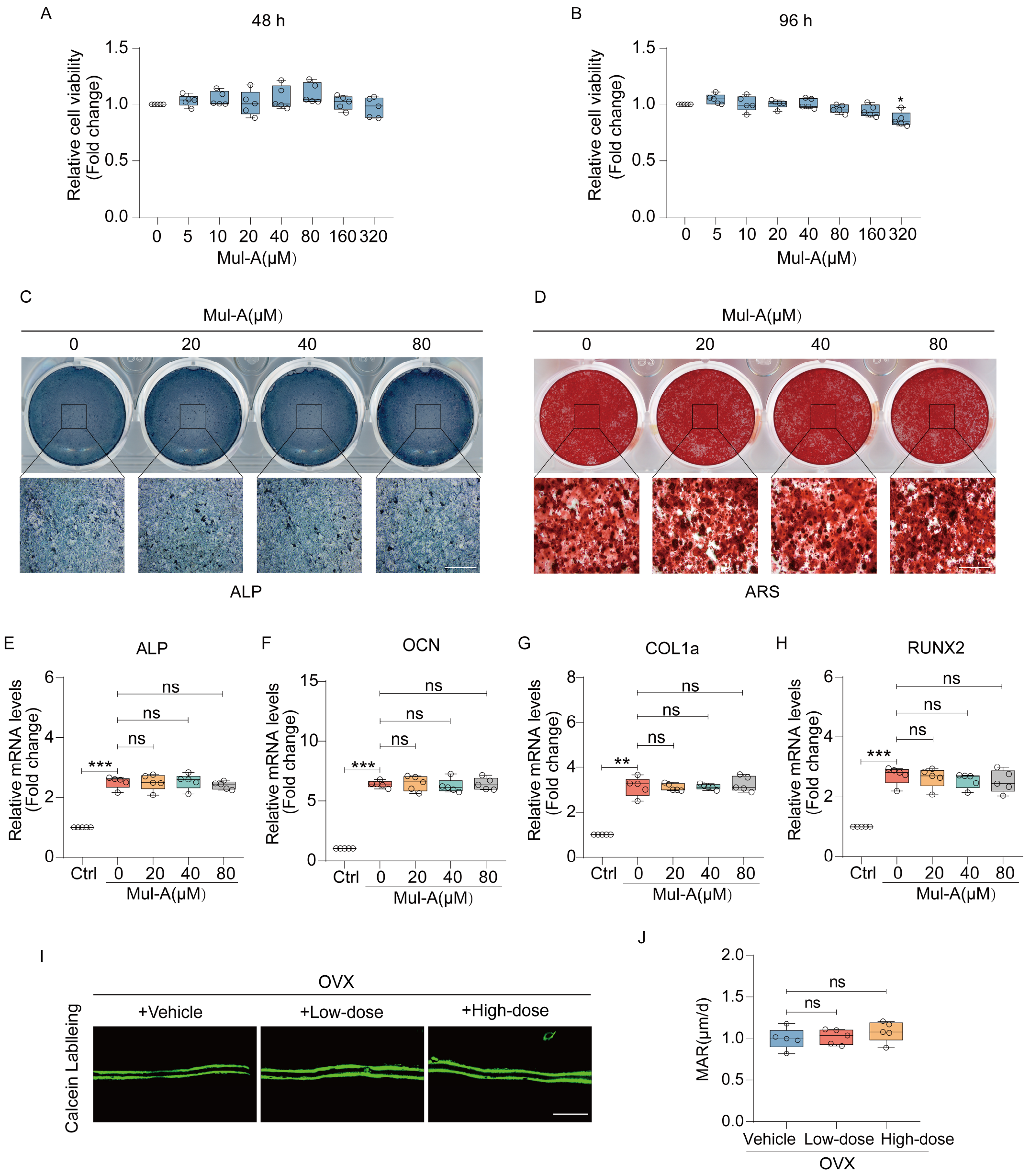

Supplementary Fig. 9 Mul-A exerts no obvious impact on osteogenesis.

(A-B) Osteoblasts were cultured with varying concentrations of Mul-A for 48 and 96 h. The cell viability assay (CCK8) was performed to assess the potential toxicity of Mul-A on osteoblasts (n = 5). (C) Osteoblasts were treated with Mul-A at concentrations of 0, 20, 40, and 80 μM for 7 days in the presence of β-glycerol sodium (10 mM) and vitamin C (100 μM). Alkaline phosphatase (Alp) staining was conducted after fixing the cells in 4% paraformaldehyde for 20 min (n = 5). Scale bar = 200 μm. (D)

Osteoblasts were treated with Mul-A at concentrations of 0, 20, 40, and 80 μM for 21 days in the presence of β-glycerol sodium (10 mM) and vitamin C (100 μM). Alizarin Red S (ARS) staining was performed after fixing the cells in 4% paraformaldehyde for 20 min (n = 5). Scale bar = 200 μm. (E-H) Osteoblasts were treated with Mul-A at concentrations of 0, 20, 40, and 80 μM for 14 days, with or without β-glycerol sodium (10 mM) and vitamin C (100 μM). Quantitative real-time PCR was employed to assess the expression of osteogenesis-related genes (n = 5). (I) In vivo, a calcein labeling assay was conducted to evaluate osteoblast activity (n = 5). Scale bar = 5 μm. (J) Quantitative analysis of the mineralization apposition rate (MAR) (n = 5). The control group was added with an equivalent DMSO. Data were presented as the median and interquartile range (IQR). *P<0.05, **P<0.01, ***P<0.001.

Supplementary tables

Supplementary tables 1. Antibody Sources and Identifiers.

Antibodies	Species	Source	Identifier	RRID	Dilutions (WB/IF)
Nfatc1	Mouse	Abcam	ab25916	AB_448901	1:1000
c-Fos	Rabbit	Abcam	ab222699	AB_2891049	1:1000
Ctsk	Rabbit	Abcam	ab187647	AB_2891139	1:1000
Trap	Rabbit	Abcam	ab191406	/	1:1000
Lc3	Rabbit	Abcam	ab192890	AB_2827794	1:1000
β-Actin	Mouse	Abcam	ab8226	AB_306371	1:10000
GAPDH	Rabbit	Proteintech	10494-1-AP	AB_2263076	1:10000
Histone 3	Rabbit	Proteintech	17168-1-AP	AB_2716755	1:5000
Atg5	Rabbit	Cell Signaling Technology	#12994	AB_2630393	1:1000
P62/Sqstm1	Rabbit	Cell Signaling Technology	#23214	AB_2798858	1:1000
Atg16l1	Rabbit	Cell Signaling Technology	#8089	AB_10950320	1:1000
Mitf	Rabbit	Cell Signaling Technology	#97800	AB_2800289	1:1000/1:200
Flag	Rabbit	Cell Signaling Technology	#14793	AB_2572291	1:1000

Supplementary tables 2. Primers Sequences

Genes	Forward Primers(5'-3')	Reverse Primers(5'-3')
NFATC1	CAGTGTGACCGAAGATACCTGG	TCGAGACTTGATAGGGACCCC
CTSK	GTTACTCCAGTCAAGAACCAGG	TCTGCTGCACGTATTGGAAGG
TRAP	CACTCCCACCCTGAGATTTGT	CATCGTCTGCACGGTTCTG
ATP6v0d2	CAGAGCTGTACTTCAATGTGGAC	AGGTCTCACACTGCACTAGGT
OSCAR	CCTAGCCTCATACCCCCAG	CGTTGATCCCAGGAGTCACAA
DC-STAMP	CGGCGGCCAATCTAAGGTC	CCCACCATGCCCTTGAACA
MMP9	GGACCCGAAGCGGACATTG	CGTCGTCGAAATGGGCATCT
ATG1	TGGAGGTGGCCGTCAAATG	CGCATAGTGTGCAGGTAGTC
ATG3	GTGAAGGCATATCTTCCGACAG	CTTGCTTTCCAGTGTAATCTCCT
ATG5	TGTGCTTCGAGATGTGTGGTT	GTCAAATAGCTGACTCTTGGCAA
ATG16L1	AACGCTGTGCAGTTCAGTCC	AGCTGCTAAGAGGTAAGATCCA
CLCN7	GACAACAGCGAGAATCAGCTC	CCAATGAGGGCACAGATAACC
BECN1	ATGGAGGGGTCTAAGGCGTC	TCCTCTCCTGAGTTAGCCTCT
VPS34	AACAACCGTGTCGCTCTTTG	GAACCATCTGCCTCCACGTTA
MITF	CAAATGGCAAATACGTTACCCG	CTCCCTTTTTATGTTGGGAAGGT
ALP	CCAACTCTTTTGTGCCAGAGA	GGCTACATTGGTGTTGAGCTTTT
COL1a	CACGCATGAGCCGAAGCTA	GGGTTTCCACGTCTCACCA
RUNX2	AGAGTCAGATTACAGATCCCAGG	TGGCTCTTCTTACTGAGAGAGG
OCN	GGCGCTACCTGTATCAATGG	GTGGTCAGCCAACTCGTCA
β-actin	GCAGGAGTACGATGAGTCCG	ACGCAGCTCAGTAACAGTCC
